# Affinity selection mass spectrometry screening discovers new COP1 peptide binders

**DOI:** 10.1039/d6cb00250a

**Published:** 2026-09-10

**Authors:** Harsha Negi, Noor Radhi, Nicolas Schiff, Sam A. Jamieson, Deanna Shea, Martin Middleditch, Robert Mullin, Renata Kowalczyk, Paul W. Harris, Peter D. Mace, Daniel Conole

**Affiliations:** a Department of Cancer Sciences, Faculty of Medical and Health Sciences, University of Auckland 85 Park Rd, Grafton Auckland 1023 New Zealand daniel.conole@auckland.ac.nz; b School of Biological Sciences, University of Auckland 23 Symonds St Auckland 1010 New Zealand; c Department of Biochemistry, University of Otago 710 Cumberland St Dunedin 9016 New Zealand; d Mass Spectrometry Hub, School of Biological Sciences, University of Auckland 23 Symonds St Auckland 1010 New Zealand; e Maurice Wikins Centre for Molecular Biodiscovery, University of Auckland Auckland 1142 New Zealand

## Abstract

Targeting E3 ubiquitin ligases with chemical probes remains a major challenge, despite their central roles in cellular regulation and disease. Constitutive photomorphogenic 1 (COP1) is an E3 ligase whose complex, adaptor-dependent biology has made its selective targeting difficult. Here, we develop a peptide-based affinity selection mass spectrometry (Pep-AS-MS) platform to discover new peptide based COP1 binders. Leveraging a mass spectrometry compatible library design, fluorescence-guided selection optimisation, and an open-source software driven target-decoy database strategy for peptide identification and quantification, we enable hit ranking from highly complex combinatorial libraries. Using this approach, we identify and validate six previously unreported COP1-binding peptides, including ligands with binding potencies comparable to the native TRIB1 motif. This work establishes a quantitative and accessible Pep-AS-MS framework for ligand discovery against challenging protein–protein interaction targets and provides new chemical tools to interrogate COP1 biology.

## Abstract 2

Screening of COP1 E3 ligase with pooled, peptide-based combinatorial libraries and affinity selection mass spectrometry revealed new peptide binder sequences for further development as cellular probes for studying COP1 biology.

## Graphical abstract

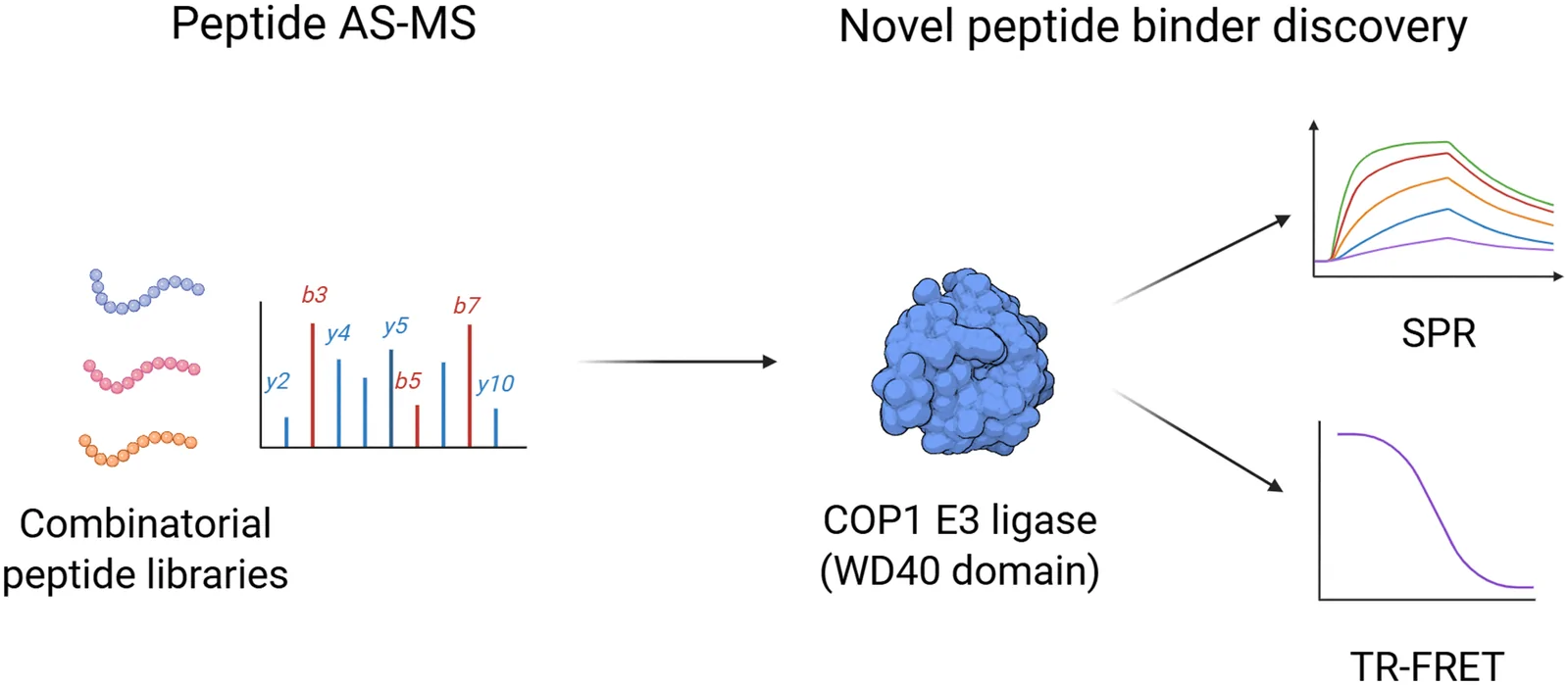

## Introduction

The post-translational modification of proteins with ubiquitin governs many important cellular processes in health and disease, including inflammation, DNA damage repair, cell signalling and protein stability.^1^ Ubiquitination of protein substrates is induced by the E1, E2, E3 enzyme cascade in the ubiquitin-proteasome system (UPS). While many different complexes exist within the UPS for protein degradation, the E3 enzyme that typically performs the final ubiquitination step is now widely considered a druggable entity for therapeutic intervention.^2^ For example, direct pharmacological inhibition of E3 enzymes can stabilise tumour suppressor substrates, or allosteric recruitment of E3s with bifunctional molecules can catalyse ubiquitination and degradation of neo-substrates, such as oncoproteins.^3^

One such important E3 ligase is constitutive photomorphogenic 1 (COP1, also known as RFWD2), which is largely conserved in both plants and humans. In humans, COP1 regulates the stability of various substrates, particularly CCAAT-enhancer-binding protein (C/EBP) transcription factors, which has implications for stem cell fate, immune cell remodelling and metabolic activity.^4–9^ COP1 has multiple modes of activity, degrading substrates either entirely by itself or in complex with substrate adaptor proteins, including TRIB1, TRIB2, DET1 and STK40.^10–14^ Whether substrates are recruited directly or indirectly, the ligand-binding pocket of COP1 is crucial because both substrates or substrate adaptors contain a common valine–proline (VP) peptidic motif that binds the pocket.

The pleiotropic and complex role of COP1 in disease means that typical target validation approaches such as gene knockdown/out are insufficient to capture these nuances in each context, giving rise to difficulties ascertaining rational therapeutic COP1 targeting strategies. As such, there is an unmet need to identify and develop potent and selective chemical probes to engage COP1 in the post-translational context, where most drugs interact, and illuminate its specific role in various diseases.

The interaction between COP1 and its substrates and adaptors has been generally well-characterised. A range of VP-motifs from transcription factor substrates bind the pocket with varying affinity.^15,16^ In humans, a TRIB1 consensus sequence containing a valine–proline (VP) motif has been shown to be important for binding the WD40 domain of COP1, and is currently the highest affinity amongst natural COP1 ligands.^20^

To date, ligand discovery for COP1 has been scarce and limited to peptides.^15,16^ There is one report of a small molecule disrupting the ubiquitination of COP1 substrate ATGL,^17^ and another of a peptide that disrupts the interaction of COP1 with its substrate p53.^18^ However neither of these works report direct COP1 target engagement.

Peptide-based affinity selection mass spectrometry (Pep-AS-MS) is an emerging pooled high-throughput screening technology for chemical probe discovery.^19–22^ Unlike its display-based pooling screening counterparts (phage and mRNA), Pep-AS-MS library members are self-encoded, which reduces the complexity of hit deconvolution from selections, and allows different binding orientations to be accessed.^23^ Moreover, Pep-AS-MS libraries are not limited to the 20 canonical amino acids, because 1 – they are built entirely using solid phase peptide synthesis (SPPS) methods, and 2 – mass spectrometry peptide sequencing is based on ionisation and molecular weight and is therefore “residue-agnostic”. More recently, Pep-AS-MS has been adapted to discover and develop macrocyclic peptides for enhanced cellular diffusion properties.^24,25^ As such, they present a powerful emerging tool to discover new COP1 binders as chemical tools to deeper understand COP1 biology in health and disease.

In this work we utilise prior knowledge about COP1 binding motifs to establish a Pep-AS-MS screening pipeline to discover new and chemically diverse peptide-based chemical probes for COP1, which may ultimately serve as new tools for studying COP1 biology.

## Results

### Design and synthesis of combinatorial peptide libraries

**Fig. 1:**
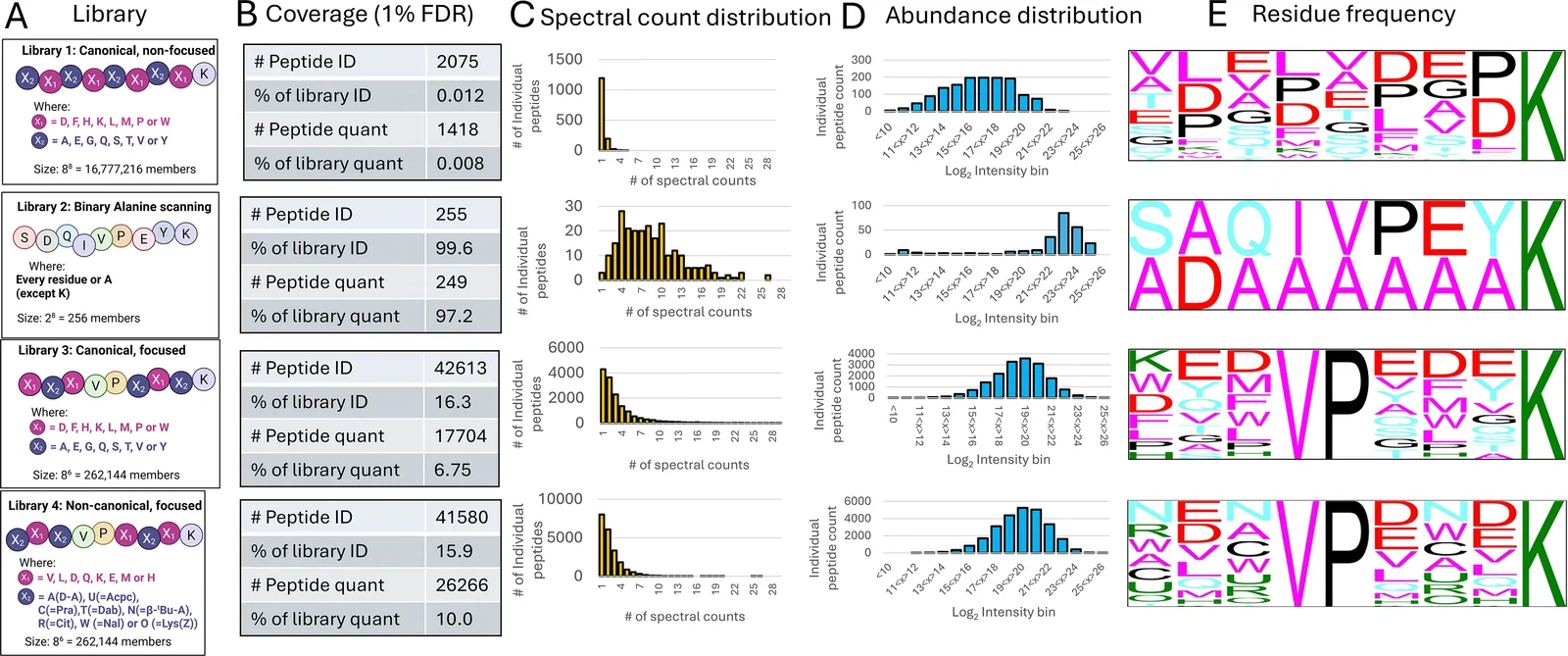
Assessment of combinatorial peptide synthesis and sequence identification performance. (A) Combinatorial peptide libraries design and total unique peptide library numbers. (B) Number and proportion of unique peptides identified and quantified (coverage) for each combinatorial peptide library, using the target-decoy database method and 1% FDR. (C) Distribution of the number of spectral counts mapped to each unique peptide ID as a measure of ID confidence. (D) Distribution of the abundance of every peptide identified and quantified from each library as a measure of combinatorial (split-pool) peptide synthesis performance. (E) Sequence logo analysis to assess the fidelity and distribution of each amino acid residue abundance at each position, based in the theoretical library design (Fig. 1A).

To explore the scope of the AS-MS utility for peptide drug discovery, we designed and synthesised 4 combinatorial libraries of varying sizes and compositions (Fig. 1A). These included an ultra-large 16.7 million member diverse, canonical amino acid library (Library 1). The other 3 remaining library designs (Libraries 2, 3 and 4) were influenced by known COP1 binding sequence from TRIB1 (SDQIVPEY), including a 256-member binary alanine-scanning library, a design first proposed by Pentelute^26^ (Library 2). Libraries 3 and 4 (262 144 members each) retained the VP motif known to be important for COP1 binding, with the difference being that the variable regions harboured either entirely canonical (Library 3) or a mix of canonical and non-canonical (Library 4) amino acids.

Influenced by seminal work from the Pentelute and Gates labs^21,22,27^ we included additional design features to optimise peptide detection by mass spectrometry for Libraries 1, 3 and 4. This entailed exclusion of isoleucine (same mass as leucine), cysteine (prevent on-resin oxidation during SPPS), asparagine (prevent deamidation side products) and arginine (poor fragmentation spectra quality) residues. The remaining canonical amino acids were partitioned into two groups of 8, to enhance the likelihood of unique mass fragments forming during MS2 ionisation. A lysine residue was fixed at the C-terminus to stabilise mass–charge states under ionisation and enhance overall peptide identification rates.

### Assessment of sequencing methods and library fidelity pre-selection

Curiously, *de novo* approaches have been used exclusively to sequence complex mixtures of combinatorially synthesised peptides.^21,22,26,28–35^ The performance of such an approach has previously been evaluated using a precision–recall analysis to determine an appropriate confidence metric cut-off for sequence identification.^22,27^ Precision measures how many of the *total* sequence IDs are correct, whereas recall measures how many library sequences are correct. In general, the more upper right-shifted the precision recall curve is, the better the performance of the *de novo* sequencing method (Fig. S1A).

We fractionated our simplest library (Binary Alanine scan, Library 2) into ten fractions and analysed them by LC-MS/MS. The raw data was then analysed by PEAKS Studio 11 *de novo* sequencing.^36^ By following the steps outlined in the previously reported precision–recall analysis,^22,27^ we could only achieve ∼40% precision and 40% recall for Library 2 (Fig. S1B).

Target-decoy based methods, where the search algorithm is given a database of true and false sequences to cross reference spectra to and ascertain peptide identification confidence, is a mainstay of endogenous proteomics analysis.^37^ Analysis of the same raw data using the target-decoy database method afforded much improved identification rates, with 98% of the total library captured at 1% false discovery rate (FDR), compared with only 41% by *de novo* sequencing (Fig. S1C and D). Although limitations exist of using such a sequencing approach for synthetic peptide ID (*e.g.* lack of scalability, limited detection of non-canonical peptides), we deemed the target-decoy database approach more suitable for our four combinatorial library designs, and therefore we used this method exclusively going forward.

With a peptide sequence identification method in hand, attention turned to analysing the contents and fidelity of our four combinatorial peptide libraries prior to any selection (Fig. 1). Very low relative rates of ID and quantification for the ultra-large, chemically diverse library 1 was observed (0.01%). Indeed, previous research groups synthesising libraries of this size report cleaving peptides from only a small number of beads prior to analysis, so that only a fractional subset of peptides could theoretically exist in the sample.^22^ This hints at the challenge associated with fully analysing such complex libraries. However, relative ID rates improved for medium sized Libraries 2 (97.2%), 3 and 4 (6.75–10.0%, Fig. 1B). This was supported by the observed increase in the proportion of multiple (*n* > 1) spectral counts for a given peptide from libraries 2, 3 and 4 compared with library 1, as evidenced by the spectral count distribution analysis in Fig. 1C.

Successful synthesis of combinatorial libraries for screening should afford approximately equal amounts of each peptide in the final mixture. To ensure our split-pool-deprotect synthesis protocol resulted in approximately equal peptide abundance, we tested this with various simple 5-mer pilot libraries whose members could be separated and quantified by low resolution LC-MS. The results showed that all peptides fell within 1 log unit of abundance, suggesting unambiguously that our split-pool-deprotect synthesis protocol was working as expected (S2A–C).

We next performed an abundance distribution analysis of all 4 unselected libraries (Fig. 1D). If successful, a narrow, symmetrical and bell-shaped abundance of distribution curve should be observed, suggesting that most peptides are present in approximately equal amounts. This was observed for libraries 2, 3 and 4, and to a lesser extent, Library 1, suggesting that by in large, our split-pool-deprotect synthesis protocol could be deployed to furnish even mixtures of library members.

Further to this, it is important that the actual library composition is true to its design, that is, that each amino acid selected for coupling is represented at the expected position in a proportion commensurate with the split number. Despite not capturing every single peptide present in Libraries 1, 3 and 4, a sufficiently high and representative number peptide IDs afforded a residue frequency analysis at each position, visualised by a sequence logo plot (Fig. 1E). With the exception of Library 1, where proline and aspartic acid appear slightly over represented at positions 2, 4, 6 and 8, each amino acid was incorporated into the combinatorial design of the library at the expected position and in the approximately expected proportion, giving confidence in library fidelity to the original design.

Despite the apparent poor fidelity of our ultra-large, unselected Library 1, we postulated that the potential for false negatives from the affinity selection were outweighed by the “up” nature of the assay, whereby hits are enriched and are therefore easier to detect after incubation with the target. Therefore, we embarked upon efforts to validate a discovery workflow for binders from COP1 affinity selections.

### Validation of an AS-MS workflow for the discovery of COP1 peptide binders

**Fig. 2:**
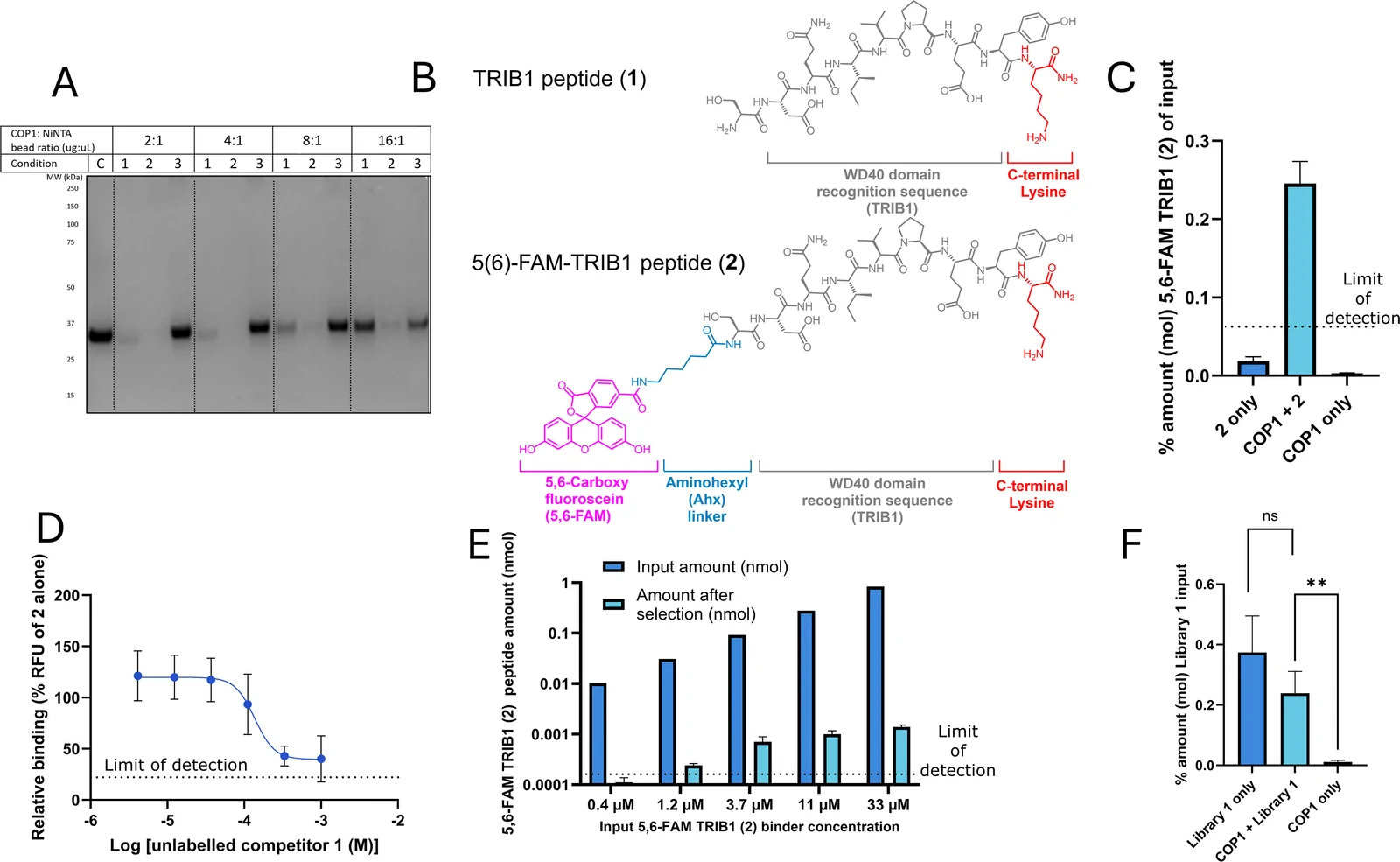
Optimisation of peptide AS-MS affinity selection protocol for COP1 using fluorescence. (A) Experiment workflow and results of SDS-PAGE of HisPur Ni–NTA magnetic affinity bead titration (5 – 0.625 µL) incubated with 2.5 µg His–COP1 target protein. Following immobilisation, bead-protein samples were washed in binding buffer and denatured with loading buffer and heating. Samples were loaded onto a gel and SDS PAGE was conducted, followed by Comassie Blue staining, and imaging. Conditiond: C – CSN5 protein (no beads); 1 – supernatant after immobilisation; 2 – supernatant after wash; 3 – heat elution. (B) Chemical structures of TRIB1 (1) and 5(6)-FAM-TRIB1 (2) peptide probes for affinity selection optimization. (C) Trial selection of 5(6)-FAM-TRIB1 (2) against COP1 with no-target and no-binder controls. (D) Competition affinity selection assay where increasing concentrations of unlabelled TRIB1 peptide (1) (1 mM to 4 µM) was selected in the presence of 10 µM 5(6)-FAM-TRIB1 (2). (E) Concentration-response affinity selections of 5(6)-FAM-TRIB1 (2) (33 µM to 137 nM) with 5(6)-FAM-TRIB1 (2) amount (nmol) detected in selection eluent. (F) Affinity selection of 20 mM Library 1 against COP1 where the selection eluent was fluorescently labelled with the pierce quantitative fluorescent peptide assay kit. Fluorescence in RFU was measured using excitation 390 nm/emission 475 nm and quantified to represent % amount of Library 1 compared to input amount into selection. Data points are mean values of triplicate results with error bars representing standard deviation. Dotted lines indicate the limit of detection of the assay as determined by a standard curve (data not shown).

We first titrated the bead:protein ratio to maximise loading of his-tagged COP1 on Ni–NTA (nickel–nitrilotriacetic acid) beads (Fig. 2A). Due to the importance of the VP motif for binding to the WD40 domain of COP1,^15,16^ we synthesised two analogues of the known COP1 binding sequence (SDQIVPEY) contained within TRIB1 to ascertain whether COP1 binding could be maintained upon bead immobilisation. These tool compounds consisted of a C-terminal lysine variant of TRIB1 (1) and a C-terminal lysine + N-terminal aminohexyl (Ahx) linked 5(6)-carboxyfluoroscein (5(6)-FAM) fluorophore TRIB1 (5(6)-FAM-TRIB1, (2)) (Fig. 2B). Calibration curves were constructed to convert fluorescent signal to 5(6)-FAM-TRIB1 (2) peptide moles, and then fluorescence was used as a fast and economical proxy for optimisation of the selection for mass spectrometry. High fluorescent signal was detected only in affinity selection conditions containing both immobilised COP1 and 5(6)-FAM-TRIB1 (2) (Fig. 2C). Moreover, the signal of 5(6)-FAM-TRIB1 (2) in the affinity selection could be outcompeted by increasing concentrations of non-fluorescent TRIB1 (1) (Fig. 2D). A range of 5(6)-FAM-TRIB1 binder (2) concentrations were assessed to inform on the relative depletion of a binder from selection input to output. The relative depletion was 99.75%, suggesting that the copy number of each library member entering the selection must be at least 400-fold higher than the limit of detection to allow for hit determination (Fig. 2E). Lastly, we utilised a fluorometric peptide assay (which fluorescently labels lysines and N-terminal amines) ascertain the amount of Library 1 retained on the beads post-selection. We showed that 500 nmol of Library 1 input resulted in measurable levels of (∼0.2 nmol, 2500-fold depletion) peptides retained on the COP1 immobilised beads after 3 washes (Fig. 2F). While no false signal was detected in the absences of the Library 1, a large fluorescent signal was detected in the absence of COP1, hinting that that non-specific bead binding would need to be accounted for in our analysis pipeline. We also investigated whether treatment of the beads with high salt (guanidine–HCl) concentrations or heat, resulted in greater peptide elution amounts post-selection, however no significant differences were observed (Fig. S3A). Performing the peptide incubation prior to elution at 25 °C performed better than at 4 °C, however (Fig. S3B). On the basis of these results, we moved forward with a 1 hour incubation at 25 °C, and a 10 minute heat-based elution (80 °C) in 50 mM PBS, as this allowed for online sample desalting, minimising samples loss before analysis.

Taken together, fluorescence-based assessment of a range of conditions allowed us to establish an optimised set of conditions to test with the more expensive and time-consuming mass spectrometry workflow. With these conditions in hand, we set about benchmarking the performance of our AS-MS workflow, using our most simple binary alanine scanning Library 2.

**Fig. 3:**
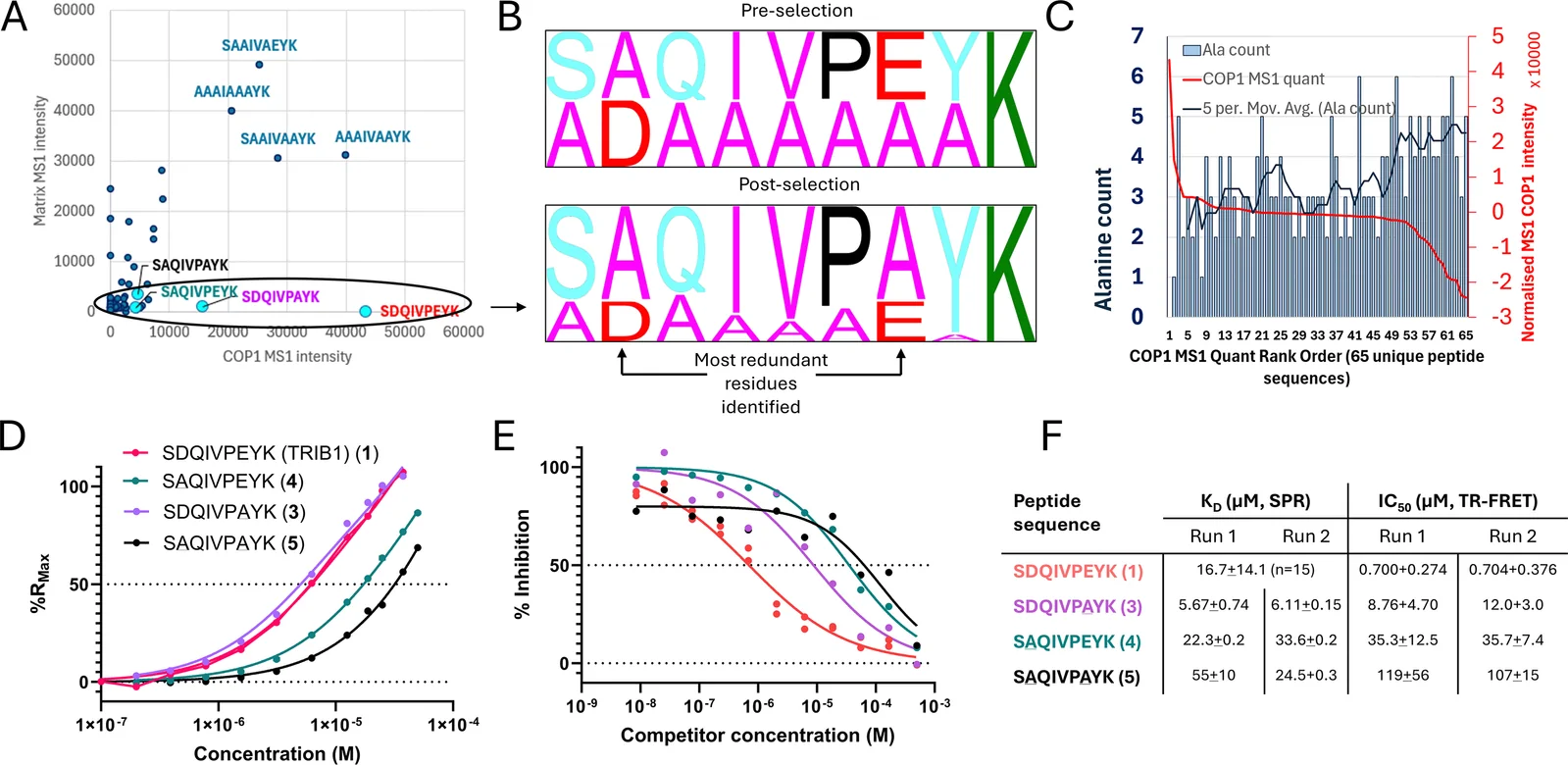
Scanning of binary alanine library for TRIB1 peptide validates the performance of affinity selection protocol and analysis pipeline. (A) *XY* scatter plot demonstrating the identity and quantity of binary alanine scanning peptides identified from an affinity selection with COP1, and NTA beads only (matrix). (B) Sequence logo (residue frequency) analysis of unique peptides that demonstrates selective binding to COP1 over NTA beads only (matrix), suggesting that substitution of aspartic acid (D) and glutamic acid (E) with alanine paid the least penalty for COP1 binding. (C) Analysis of Alanine count per peptide (blue bars) *versus* a rank ordering of COP1 binding (red line) demonstrated the expected inverse correlation. Representative concentration response curves for SPR (D) and competitive TR-FRET assays (E) confirmed the structure–activity relationships observed in the affinity selection results when replacing aspartic acid (D) and glutamic acid (E) on TRIB1 peptide (1). (F) Binding (SPR, *n* ≤ 2) and inhibition (TR-FRET, *n* ≤ 2) constants for alanine substitutions for aspartic acid (D) and glutamic acid (E) residues on TRIB1 peptide (1).

The input library copy number, that is the average amount of each library present in each selection, is an important parameter for obtaining measurable signal by mass spectrometry and therefore identifying peptide binders. We anticipated that this number should be generalisable across different library sizes, however the literature^21,31,35^ suggests a wide range of acceptable input library copy numbers (anywhere from 10 fmol to 20 pmol per member). This suggests that library size may well be important, as well as the nature and concentration of the target and the tag/bead system being used. Therefore, we performed a titration of Library 2 concentration against a constant concentration of COP1 immobilised to NTA beads to identify the optimal library copy number for our specific affinity selections. The results suggested that 100 pmol per member was optimal, as >30 out of the total 256 peptides were identified and quantified in both matrix only and COP1 immobilised selection conditions (Fig. S4). An *XY* plot of the quantification data of these identified peptides (*Y* – Matrix, *X* – COP1) showed, as expected, the most selective COP1 peptide binder was TRIB1 (1) (SDQIVPEYK) (Fig. 3A). Curiously, single alanine substitutions at the glutamate (SDQIVA̲YK, 3) and aspartate (SA̲QIVPEYK, 4), and a double substitution at the two acidic residues (SA̲QIVPA̲YK, 5) were the next most selective COP1 binders (Fig. 3A). Residue frequency analysis of all the peptides that showed some extent of COP1 selective binding (as evidenced by positioning along the bottom of the *x*-axis) suggested that these acidic residues (E and D) were the least critical for retaining TRIB1 peptide binding potency to COP1 (Fig. 3B). This is inconsistent with a conventional single alanine scan for TRIB1 reported in the literature, which suggests the tyrosine (Y) is required the least for COP1 binding.^16^ This suggests the holistic scanning provided by AS-MS evaluation of all the possible alanine substitution combination at the same time offers new insight into peptide SAR.

Overall affinity selection performance was determined by the identification of an expected reverse correlation between normalised COP1 binding (as measured by COP1 MS intensity minus Matrix MS1 intensity) and alanine count for each peptide (Fig. 3C). Individual resynthesis, purification and *in vitro* binding evaluation of TRIB1 peptide 1 and its active alanine substituted analogues (SDQIVPA̲YK 3, SA̲QIVPEYK 4, and SA̲QIVPA̲YK, 5) by SPR (Fig. 3D and F) and TR-FRET (Fig. 3E and F) demonstrated a potency ranking and range that was consistent with that observed in the raw quantitative affinity selection data, with these alanine substitutions only having minor effects on COP1 binding (Fig. 3A). These results gave us confidence to test our affinity selection workflow against our more complex and larger combinatorial peptide Libraries 1, 3 and 4.

### Affinity selection of COP1 with more complex libraries

To understand the performance of our AS-MS workflow with more complex libraries, our Library 1 (150 fmol per member, which was the maximum copy number that could be achieved for 16.7 million members with complete aqueous solubility) was incubated with a bead only control, and COP1 at two different bead loadings (5 and 10 µg), and those same conditions including a spike-in of TRIB1 peptide 1 at either (1 or 1000-fold the average concentration of each library member) were evaluated (Fig. S3B). When including the TRIB1 peptide (1) in our peptide database for peptide-spectrum matching of raw spectra obtained from the affinity selections, the TRIB1 peptide (1) emerged as a strong hit across all three technical replicates of the affinity selection samples containing COP1 and 1000-fold spiked-in TRIB1 peptide (1) (Fig. S3B). However, some false positive TRIB1 (1) identifications emerged in some negative control samples (Fig. S3B). Further increases in target loading and more stringent “match-between run” or (MBR) settings on the Fragpipe search software lead to strong identification and enriched quantities of TRIB1 peptide (1) only in beads loaded with COP1 (20 µg), and not in bead only or non-spiked in negative controls (Fig. 3A). This suggested that our affinity selection and MS-based peptide ID and quantification workflow can detect true hits from complex mixtures, albeit with a high excess of a known binder (TRIB1, 1).

**Fig. 4:**
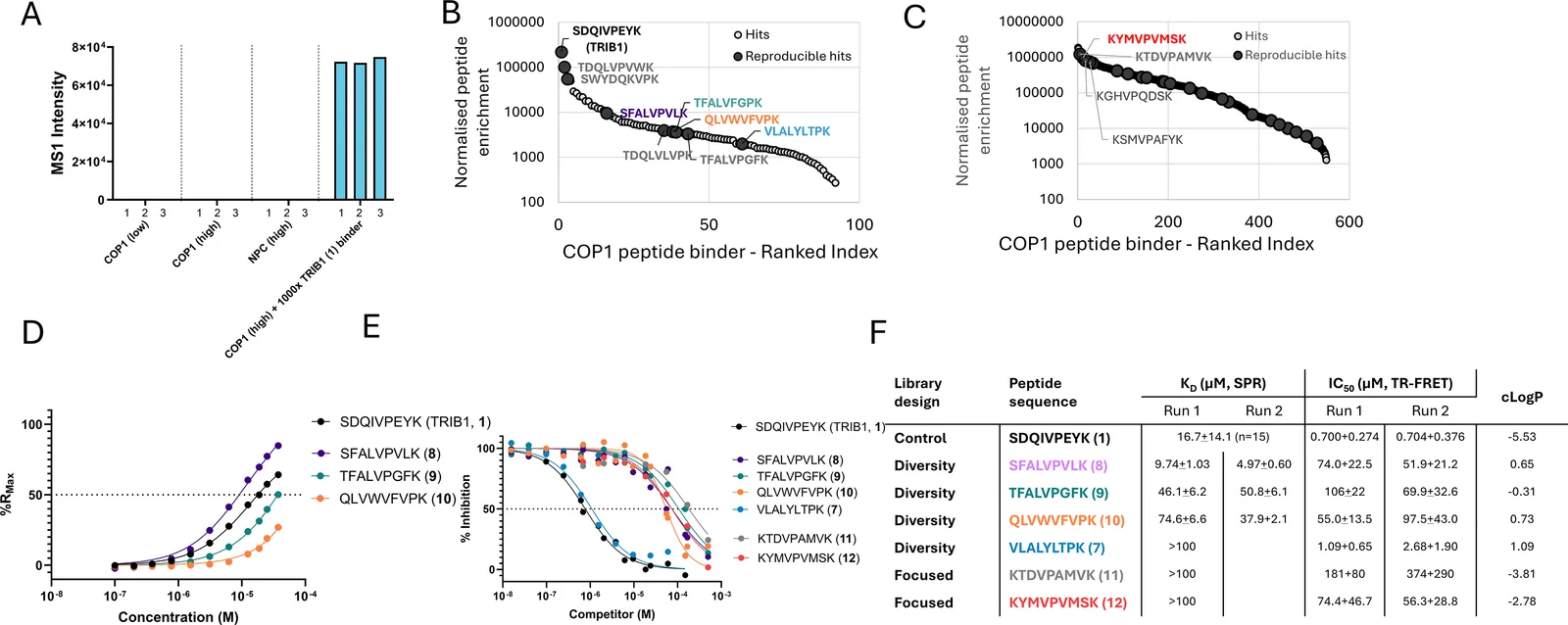
Affinity selection with larger libraries leads to identification and validation of potent, new and lipophilic COP1 peptide binders. (A) Spike in (1000-fold excess relative to average abundance of individual library members) experiments demonstrate that the TRIB1 peptide (1) is only identified and enriched when both the peptide and the target (COP1) (high = 20 µg loaded, low = 10 µg loaded) are present in the affinity selection (NPC = No Protein Control). Waterfall plot ranking of hits determined from a representative affinity selection with the diverse, 16.7 million member library (B) and the canonical focused 262 000 member library (C). Hits were determined as peptides with positive quantitation scores (determined by area under the curve of the MS1 peak) after summing the individual quantities for COP1 conditions and subtracting the sum of the individual quantities for matrix only conditions. Reproducible hits (larger, black dots) were determined by their identification across *n* = 2 biological replicates. Coloured peptides represent those that validated in subsequent orthogonal binding assays. Representative concentration response curves for SPR (D) and competitive TR-FRET assays (E) for peptides that were individually resynthesized and demonstrated <500 µM affinity/inhibition constants. (F) A summary of the binding (SPR, *n* ≤ 2) and inhibition (TR-FRET, *n* ≤ 2) constants for peptides that were individually resynthesized and demonstrated <500 µM affinity/inhibition constants.

We then set out to triage COP1 peptide binders from this affinity selection. While an initial statistics-based students *t*-test to detect significant differences between groups was desired, a large number of missing values (*i.e.* a peptide was detected in one technical replicate within a group but not the other) precluded this approach. Instead, the MS intensity values of peptides confidently identified (1% FDR) from each COP1 loaded condition were summed, normalised by subtracting the MS intensity values for those peptides for the bead only control, and ranked (Fig. 4B and Fig. S5B). As such, peptides that were quantified in more than one COP1 replicate received a higher score. Selection conditions containing COP1 only and COP1 with TRIB1 peptide (1) spike-in were summed together, as it was assumed that the concentration of TRIB1 peptide in the spike-in (1000× fold greater than the average member concentration) was not sufficient to limit the detection of all competitive binders. As such, the binder candidate sequence list most likely contains both active site and allosteric binders, however we did not investigate this further. Applying our Fragpipe based hit triage workflow, 92 hits were identified, with 2 of these hits (TDQVPVWK, 6 and VLALYLTPK, 7) also present in a subsequent repeat affinity selection (Fig. 4B and Fig. S5A). Many of these peptides contained the VP-motif, which is consistent with the importance of this motif for binding to the WD40 domain.^15,16^ We selected 8 of these hits for individual resynthesis and binding validation on the basis of containing a VP motif, and structural diversity (Fig. 4B). Of these 8, three peptides showed quantitative COP1 binding by SPR, with the rest either showing no detectable binding (KD > 100 µM), or low binding affinity obscured by non-specific binding events at high peptide analyte concentrations (Fig. S7D–F). On-target binding of the COP1 substrate-binding pocket was confirmed for four hits using a TR-FRET. In particular, two sequences, namely SFALVPVLK (8), as measured by SPR (Fig. 4D–F and Fig. S5C) and VLALYLTPK (7), as measured by TR-FRET (Fig. 4E, F and Fig. S5E) demonstrated similar or enhanced potency compared to TRIB1 (1). Poor aqueous solubility of peptide 7 precluded its accurate binding measurements by SPR (data not shown). VLALYLTPK (7) was also tested by TR-FRET using a 10-fold excess of Tween-20, which has been shown to prevent compound aggregation that is known to cause false positives in binding assays (Fig. S7B).^38^ Only tested at *n* = 1 technical replicates, the variability of this measurement precluded any strong conclusions, however VLALYLTPK (7) inhibited (5(6)-FAM-TRIB1 (2) binding to a similar extent to that of TRIB1 (1) suggested peptide aggregation was not driving a false positive TR-FRET result. Although not by design, the most potent binders showed marked increases in *c* log *P* (>5 *c* log *P* unit change) compared with TRIB1 (1) (Fig. 4F). We were also able to show by immobilisation of an unrelated protein (CSN5) on the reference flow cell that the SPR-based binding of SFALVPVLK (8) is specific to COP1, and not due a non-specific protein binding and/or aggregation effect on the SPR sensor chip due to increased lipophilicity (Fig. S7A).

We also performed the same affinity selections and analysis workflows to identify COP1 binding hits for our VP focused canonical Library 3 (Fig. 4C and Fig. S5B) and non-canonical Library 4 (Fig. S6A and B). From canonical VP focused Library 2, we identified 4 peptides that emerged as hits from repeat affinity selections, and resynthesized and evaluated the binding of these individually. Two of these (KTDVPAMVK, 11 and KYMVPVMSK, 12) showed weak competitive displacement (IC_50_ = ∼50–400 µM) of 5(6)-FAM-TRIB1 (2) by TR-FRET (Fig. 4E, F and Fig. S5D). Regarding non-canonical VP focused library 4, only one hit (coded as NEUVPVWEK (13), emerged across the two replicate affinity selections (Fig. S6A and B). The hit is amino acid coded as NEUVPVWEK (13), however, the X_2_ positions of the library design are actually non-canonical amino acids (see Fig. S6C) therefore the actual structure is displayed in Fig. S6D. Resynthesis and binding evaluation of peptide 13 by SPR and TR-FRET did not lead to any appreciable COP1 binding, however (Fig. S6E and F).

## Discussion

In this work, we use peptide based AS-MS to screen for novel peptide binders for COP1, an E3 ligase important in cancer and inflammation. We discover and validate 6 new COP1 peptide binders, including two structurally distinct sequences exhibiting similar binding potency to the known COP1 binder, TRIB1 (1), but with much higher lipophilicity. While enhanced peptide lipophilicity is beneficial for their cellular permeability, it is also common that lipophilic peptides can suffer from solubility and aggregation issues. While we do perform some studies towards the understanding of whether our hit peptides exhibit non-specific or aggregative, colloid formation effects that might affect their binding readouts, further studies would need to be undertaken to confirm this, such as dynamic light scattering.^38^ Future studies would also need to confirm in cell-based assays whether the increased lipophilicity of hit peptides 7 and 8 actually enhances their cellar permeability.

To our knowledge, this is the first report of a synthetic peptide AS-MS workflow that incorporates an open source (Fragpipe) target-decoy, database search method for identifying and quantifying peptide binders. Pentelute and others rely on a *de novo* sequencing method for this, however in our hands we found this method to be sub-optimal, leading to lower numbers of peptide IDs (as determined by a ALC score > 90) than target-decoy database methods (as determined by a 1% FDR) (Fig. S1).

Target decoy-based methods are a relatively standard approach in endogenous bottom-up proteomics approaches, so it is surprising that this method has never been reported before. A reason for this may be that for complex synthetic peptide libraries, this method lacks scalability, as very large sequence database FASTA files will take extremely long periods to load into the search software. It may also be that the default database search parameters exclude any peptide side-products and truncates, which may bias the results and lead to false positives, however this could be addressed by altering the search parameters. Nonetheless, we found it to perform well, especially for our mid-sized (10^5^ unique members) libraries, and in terms of assessing pre-selection library fidelity (Fig. 1). Independent of the peptide ID method used (*de novo* or database), the incorporation of non-canonical amino acids presents similar challenges, as library diversity is limited by the number of single letter codes that exist in the search software and algorithms used (*n* = 26). Nonetheless, we were able to show the 4 of the out 8 variable positions on other combinatorial 9-mer peptide library design could be replaced with non-canonical amino acids, and this leads to similar ID rates to that of entirely canonical peptide libraries. We would welcome a solution from the inventors of Fragpipe or other platforms to extend the number of codes for deeper non-canonical amino acid sampling.

While *de novo* sequencing methods have been extensively utilised for the discovery and validation of new peptide binders, commensurate quantitation data, coupled with a disclosed hit triage pipeline, has been missing. We believe this is the first report of a target-decoy database method with comprehensively matched quantitative data for each identified peptide, which allows for rational hit ranking and consequently minimal labour expense for hit validation.

Recently, Brown *et al.*^30^ reported a peptide hit triage method termed PyBinder, which scores peptide features based on target selectivity and concentration-dependent enrichment (CDE). The precursor ions for peptide features that meet the target selectivity and CDE criteria are then used as a database for instructing the mass spectrometer to fragment these ions upon a second sample run. This leads to an increase in the number of peptide binders identified. While an elegant solution to the complexity of affinity selection eluents and the “missing value” problem of AS-MS, it requires samples to be run again, which costs more instrument time and more selection eluent, which may in turn cost more precious recombinant target material. We believe that the well-established target-decoy database-based search method, coupled with a similar concentration and target dependent experimental design, should afford similar results, but with high confidence peptide identification and quantification at the same time, providing a more straightforward solution for hit triage and uptake of this technology by new users. Moreover, our pre-selection library fidelity analysis (Fig. 1) and affinity selection optimisation using fluorescent probes (Fig. 2) provides a high quality, low cost and generalisable workflow for establishing peptide-based AS-MS against new targets, especially where a low affinity peptide fragment binder sequence is already known.

Despite demonstrating an impressive 30% confirmation rate (6/17) from the peptides that selected for hit validation, the average potency of these hits was low. This could be attributed to the tractability of the target, as COP1 has been shown to be a challenging to ligand by DEL screening, compared other WD40 repeat (WDR) proteins.^39^ It was also interesting to see that the most potent hits came from diversity-based Library 1, rather than VP-focused libraries 3 and 4, suggested deep chemical sampling is critical for generating new chemical matter for targets.

Peptide AS-MS is an emerging new drug screening technology with immense potential for improvement. An intermediate hit validation component, such as an array-based binding assay, would serve as a powerful tool to test most if not all of the hundreds of hits identified in these experiments.^40^ Moreover, a rapidly confirmed peptide hit list could serve as a powerful tool to inform further focused combinatorial library design and/or machine learning approaches that take into consideration a multitude of other factors, such as optimal physicochemical properties for cellular diffusion, as well as target-specific structure biology.

## Conclusions

We report a peptide-based affinity selection mass spectrometry (Pep-AS-MS) workflow for the discovery of ligands targeting the E3 ubiquitin ligase COP1, a regulator of transcription factor stability with complex roles in disease. By combining careful library design, fluorescence-guided selection optimisation and validated mass-spectrometry-based identification, we demonstrate that Pep-AS-MS as a powerful tool for preparing and screening focused and highly complex combinatorial peptide libraries.

A central advance of our study is the implementation of an open-source, target-decoy database search strategy for synthetic peptide identification and quantification. Compared with *de novo* sequencing approaches, this method afforded improved peptide identification rates and matched quantitative data, enabling rational hit ranking and efficient triage from complex selections.

Using this platform, we identified and validated six previously unreported COP1-binding peptides, including sequences exhibiting binding potencies comparable to the native TRIB1 motif. These ligands provide valuable starting points as chemical tools for probing COP1 biology beyond genetic approaches and in a post-translational context.

This work establishes a quantitative and accessible Pep-AS-MS framework that is generalisable for ligand discovery against challenging protein–protein interactions with limited structural biology available.

## Conflicts of interest

There are no conflicts to declare.

## Supplementary Material

CB-OLF-D6CB00250A-s001

## Data Availability

The data supporting this article have been included as part of the supplementary information (SI). Supplementary information is available. See DOI: https://doi.org/10.1039/d6cb00250a.
