# Supplementary material for: Affinity selection mass spectrometry screening discovers new COP1 peptide binders
Source: RSC Chem Biol. 2026 Sep 10;7(11):2225–33. doi: 10.1039/d6cb00250a (PMC13586983; doi:10.1039/d6cb00250a)
Supplement: Supplementary file 1 [file CB-007-D6CB00250A-s001.pdf]

Supplementary Information (SI) for RSC Chemical Biology.
This journal is © The Royal Society of Chemistry 2026

Supplementary materials, methods and information for “Affinity selection mass spectrometry screening discovers new and more lipophilic COP1 peptide binders”

Harsha Negi[a,b,e], Noor Radhi[a], Nicolas Schiff[b], Sam A. Jamieson[c], Deanna Shea[d], Martin Middleditch[d], Robert Mullin[b], Renata Kowalczyk[b,e], Paul W. Harris[b,e], Peter D. Mace[c,e], and Daniel Conole*[a,e]

a. Department of Cancer Sciences, Faculty of Medical and Health Sciences, University of Auckland, 85 Park Rd, Grafton, Auckland, 1023, New Zealand

b. School of Biological Sciences, University of Auckland, 23 Symonds St, Auckland, 1010, New Zealand

c. Department of Biochemistry, University of Otago, 710 Cumberland St, Dunedin, 9016, New Zealand

d. Mass Spectrometry Hub, School of Biological Sciences, University of Auckland, 23 Symonds St, Auckland, 1010, New Zealand

e. Maurice Wikins Centre for Molecular Biodiscovery, University of Auckland, Auckland 1142, New Zealand

Table of Contents

1. Materials and Reagents: ... 3

2. Synthesis of Peptides and Peptide Libraries ... 3

  2.1 Peptide Synthesis ... 3

    General procedure for manual solid phase peptide synthesis (SPPS) ... 4

    2.1.2 General procedure for flow solid phase peptide synthesis. ... 5

    2.1.3 General procedure for Split and Pool SPPS ... 5

  2.2 Design of combinatorial libraries ... 6

    2.2.1 Pilot 5-mer library ... 6

    2.2.2 Binary alanine scanning 256 member library or Library 2 ... 6

    2.2.3 VP focused canonical amino acid 262144-member library or Library 3 ... 6

    2.2.4 VP focused non-canonical amino acid 262144-member library or Library 4 ... 7

  2.3 Crude Library Recovery and Yield Analysis ... 7

    2.3.1 Mass Recovery of Library 2-4 ... 7

  2.4 Desalting and Fractionation of Libraries ... 7

3. Characterisation of Synthesised Peptides ... 7

  3.1 SDQIVPEYK (Trib1-Lys)-NH_2 (1) ... 7

  3.2 5(6)-FAM-Ahx-SDQIVPAYK-NH_2 (5(6)-FAM-TRIB1-Lys) (2) ... 8

  3.3 SDQIVPAYK -NH_2 (3) ... 9

  3.4 SAQIVPEYK-NH_2 (4) ... 10

  3.5 SAQIVPAYK-NH_2 (5) ... 10

  3.6 TDQLVPVWK-NH_2 (6) ... 11

  3.7 VLALYLTPK-NH_2 (7) ... 12

3.8 SFALVPVLK-NH_2 (8) 12
3.9 TFALVPGFK-NH_2 (9) 13
3.10 QLVWVFVPK-NH_2 (10) 13
3.11 KTDVPAMVK-NH_2 (11) 14
3.12 KYMVPVMSK-NH_2 (12) 15
3.13 NEUVPVWEK-NH_2 (13) 16
3.14 KSMVPAFYK-NH_2 (14) 16
3.15 KGHVPQDSK-NH_2 (15) 17
4 Development of the COP1 Affinity Selection Platform 18
4.1 Recombinant WD40 domain COP1 protein production 18
4.2 Optimisation of COP1 Protein Loading 18
4.3 Optimisation of the affinity selection using 5(6)-FAM-TRIB1-Lys 19
4.4 Pilot Affinity Selection Using Library 1 19
5. Affinity Selection Mass Spectrometry Screening 20
5.1 Affinity selection with Library 1 20
5.2 Affinity selection with Library 2 20
5.3 Affinity selection with Libraries 3 and 4 20
6. Identification of Library Hits and analysis 21
6.1 LC-MS/MS analysis of Library 1-4 21
6.2 Precision-recall analysis 21
6.3 Data Processing and Analysis of Peptide Libraries with FragPipe 21
6.4 Post-sequencing analysis 22
6.5 Hit-Triage analysis 22
7. Hit Validation 22
7.1 Surface Plasmon Resonance (SPR) assay of selected Hit peptides 22
7.2 Time-resolved Fluorescence resonance energy transfer (TR-FRET) assay of selected Hit peptides 23
References 24

1. Materials and Reagents:

Fmoc-Ser(tBu)-OH, Fmoc-Lys(Boc)-OH, Fmoc-Ala-OH, Fmoc-Glu(OtBu)-OH, Fmoc-Gly-OH, Fmoc-Gln(Trt)-OH, Fmoc-Val-OH, Fmoc-Tyr (tbu)-OH, Fmoc-Asp(OtBu)-OH, Fmoc-Leu-OH, Fmoc-His(Trt)-OH, 1-[bis(dimethylamino)methylene]-1H-1,2,3-triazolo[4,5-b]pyridinium 3-oxid hexafluorophosphate (HATU) were purchased from Aaptec LLC (Louisvelle, KY, USA). Fmoc-L-propargylglycine (Fmoc-Pra-OH), Fmoc-D-3-(2-Naphthyl)-alanine, Fmoc-Lys(Z)-OH, Fmoc-D-citrulline (Fmoc-D-Cit-OH), N-alpha-Fmoc-N-gamma-Boc-D-diaminobutanoic acid (Fmoc-D-Dab(Boc)-OH), 1-Hydroxy-6-chlorobenzotriazole (6-ClHoBt), Triisopropylsilane (iPr3SiH), 5(6)-carboxyfluorescein (5(6)-FAM) and 6-aminohexanoic acid were purchased from AK Scientific, Inc (Union City, CA, USA). Fmoc-D-Ala-OH was purchased from Auspep Pty Ltd (Tullamarine, VIC, Australia). Fmoc-Thr(tBu)-OH, Fmoc-Phe-OH, Fmoc-Met-OH, Fmoc-Pro-OH, Fmoc-Trp(Boc)-OH, O-(6-Chlorobenzotriazol-1-yl)-N,N,N',N'-tetramethyluronium hexafluorophosphate (HCTU) were purchased from CS Bio (Shanghai) Ltd (Shanghai, China). Acetic anhydride, dimethyl formamide (DMF), dichloromethane (CH2Cl2), piperidine, formic acid, diethyl ether, trifluoroacetic acid (TFA) was purchased from ECP Ltd (Auckland, New Zealand). N,N-diisopropylethylamine (iPr2NEt), N-methylmorpholine (NMM), 2,4,6-trimethylpyridine (collidine), 1,2-ethanedithiol (EDT) and Bovine Serum Albumin (BSA) were purchased from Sigma Aldrich (St. Louis, MO, USA). Acetonitrile, HPLC grade (MeCN) and HisPurTM Ni-NTA magnetic beads and Tween 20 were purchased from Thermo Fisher Scientific (Hampshire, NH, USA). Fmoc-1-amino-1-cyclopropan carboxylic acid, 2-(4-(((((9H-fluoren-9-yl)methoxy)carbonyl)amino)(2,4-dimethoxyphenyl)methyl)phenoxy)acetic acid (Fmoc-Rink amide) and N,N-diisopropylcarbodiimide (DIC) were purchased from Aaron Chemicals, LLC (San Diego, CA, US). TentaGel S-NH_2 resin 90 micron (0.24 mmol/g) was purchased from Rapp Polymere GmbH (Tuebingen, Germany). HBSTE buffer (10X), Regeneration kit intensive (K RIN-50I), Buffer kit for NiHC sensor chips (K NI-50I), SPR sensor chip (NiHC 1000M) were purchased from XanTec bioanalytics (Düsseldorf, Germany).

2. Synthesis of Peptides and Peptide Libraries

2.1 Peptide Synthesis

Manual Fmoc/tBu SPPS was used for the assembly of SDQIVPEYK or Trib1-Lys (1), 5(6)-FAM-Ahx-SDQIVPEYK or 5(6)-FAM-TRIB1-Lys (2), SFALVPVLK (8), TFALVPGFK (9), QLVWVFVPK (10), VLALYLTPK (7), TDQLVPVWK (6).
Flow Fmoc/tBu SPPS was used for the assembly of SDQIVPAYK (3), SAQIVPEYK (4), SAQIVPAYK (5), KTDVPAMVK (11), KYMVPVMSK (12), KGHVPQDSK (15), KSMVPAFYK (14), NEUVPVWEK (13)
Split-pool Fmoc/tBu SPPS was used for the assembly of Pilot 5-mer library and Library 1-4. TentaGel S NH_2 90 um aminomethyl resin (0.24 mmol/g) preloaded with Fmoc-Rink amide linker, on either 0.1 or 0.4 mmol scale was used as a solid support.
When required, the Kaiser test[1] was used to monitor completeness of Fmoc-AA-OH couplings and Nα-Fmoc protecting group removal.

For Fmoc-Rink amide linker attachment, a solution of Fmoc-Rink amide (3 equiv.), DIC (3 equiv.) and 6-Cl-HOBt (3 equiv.) in DMF/CH_2Cl_2 (v/v, 2 and 5 ml in total, for 0.1 and 0.4 mmol scale, respectively) was added to the pre-swollen resin (DMF/CH_2Cl_2 (v/v, 2 and 5 ml in total, for 0.1 and 0.4 mmol scale, respectively, 30 min). After agitating the mixture for 2 h at room temperature, the solution was drained, and the resin washed with DMF (3 x 5 mL).

After the last step of synthesis, the peptidyl resin was washed thoroughly with CH_2Cl_2 and dried under the flow of nitrogen. Resin cleavage with simultaneous removal of the amino acid side-chain protecting groups was undertaken by incubating the resin in TFA/iPr$_3$SiH/H_2O/EDT (v/v/v/v; 94/1/2.5/2.5) cleavage cocktail (5, and 15 mL in total, for 0.1 and 0.4 mmol scale, respectively) for 3 h at room temperature. The crude peptides were precipitated and triturated with cold diethyl ether, isolated (centrifugation), dissolved in 50% MeCN (aq) containing 0.1% TFA and lyophilized, prior their final purification by RP-HPLC (peptides 1-15), or used as synthesised (Libraries 1-4, Pilot 5-mer library).

Analytical reverse-phase high-performance liquid chromatography (RP-HPLC) was performed on either a Waters (Waltham, MA, USA) Alliance analytical HPLC equipped with an Agilent (Santa Clara, CA, USA) TC C18 column, or Shimadzu (Kyoto, Japan) Prominence LC-20AP Liquid Chromatography modular system equipped with SPD-M20A Prominence diode array detector equipped with a Waters (Milford, MA, USA) XTerra MS C18 column (125 Å, 5 µm, 4.6 x 150mm), operated at room temperature, with chromatograms recorded at 214 or 280 nm, at a flow rate of 1 mL/min. Analytical RP-HPLC gradient employed were adjusted according to eluting profiles of the synthesized peptides. A linear gradient of 1% B to 61% B (ca. 3% B per min) was used for peptide **1**;[1] 1% B to 61% B (ca. 1%B per min) was used for peptides **6, 7, 8, 9**; 5% B to 95% B (ca. 3%B per min) was used for peptides **2**, and**10**; 1% B to 41% B (ca. 1%B per min) was used for peptides **3**, **11**, and **13**, and 1% B to 21% B (ca. 1%B per min) was used for peptides **4,5,12,14**, and **15**.

Semi-preparative RP-HPLC was performed on a Waters 1525 Binary HPLC pump equipped with a Waters 2489 UV/visible detector using a Phenomenex Gemini-NX C18 (110 Å, 10 µm, 10 mm × 250 mm), or Waters XTerra MS C18 OBD Prep column (125 Å, 10 µm, 10 x 250mm), at a flow rate of 5 mL/min. Gradient systems used for semi-preparative RP-HPLC were adjusted according to the elution and peak profiles obtained from the analytical RP-HPLC chromatograms. A linear gradient of 1% B to 61% B (ca. 0.5%B per min) was used for peptides **1**, **6, 7, 8, 9,** and **10**; 5% B to 95% B (ca. 1%B per min) was used for peptide **2**; 1% B to 41% B (ca. 0.5%B per min) was used for peptides **3-5, 11-14** , and 1% B to 41% B (ca. 0.25% B per min) for peptide **15**. Fractions were collected, analysed by either RP-HPLC or ESI-MS, pooled and lyophilised.
For both analytical and semi-preparative RP-HPLC, solvents used were 0.1% TFA in MQ H_2O (solvent A), and 0.1% TFA in MeCN (solvent B).

High resolution mass spectra (HRMS) were obtained using the Bruker micrOTOF-Q spectrometer operating at a nominal accelerating voltage of 70 eV. ESI- MS spectra were acquired on an Agilent Technologies (Santa Clara, CA) 1260 Infinity LC equipped with an Agilent Technologies 6120 Quadrupole mass spectrometer. Solvents used were 0.1% formic acid in MQ H_2O (solvent A), and 0.1% formic acid in MeCN (solvent B).

General procedure for manual solid phase peptide synthesis (SPPS)

Peptides **1, 2, 6-10** were synthesized using manual Fmoc/*t*Bu SPPS (0.1 mmol) and using a fritted glass reaction vessel.

---

[1] LC-MS spectrum was acquired for purified peptide **1** using an Agilent Technologies (Santa Clara, CA) 1260 Infinity LC equipped with an Agilent Technologies 6120 Quadrupole mass spectrometer, and analytical Agilent C3, (3.5 µm; 3.0 × 150 mm) column at a flow rate of 0.3 mL/min.

The swelling of Fmoc-Rink amide-TentaGel S NH_2 90 um aminomethyl resin was performed using DMF (5 mL), for 30 min, prior the synthesis N^{α}-Fmoc protecting group removal was performed using 20% piperidine in DMF, containing 5% formic acid (v/v, 3 mL) for 10 min, followed by the second deprotection step using fresh reagents (10 min). The peptidyl-resin was then washed with DMF (5 × 5 mL).
For the coupling of amino acids, a mixture of Fmoc-AA-OH (5.0 equiv., 0.5 mmol), HCTU (4.75 equiv., 0.48 mmol) and NMM (10.0 equiv., 1.0 mmol) in DMF (2 mL) was added to the resin and reaction vessel shaken for 30 min. 6-aminohexanoic acid, and 5(6)-FAM (5.0 equiv., 0.5 mmol), were coupled using DIC (5.0 equiv., 0.5 mmol) and 6-Cl-HOBt (5.0 equiv., 0.5 mmol), in DMF (2 mL), for 2 h. The resin was drained and washed with DMF (3 x 5 mL). After the coupling of 5(6)-FAM, the peptidyl-resin was treated with 20% piperidine in DMF, containing 5% formic acid (v/v, 3 mL) for 5 min, then drained and washed with DMF (3 × 5 mL). This procedure was repeated 10 times.

2.1.2 General procedure for flow solid phase peptide synthesis.

Peptides **3-5 and 11-15** were synthesized using flow Fmoc/tBu SPPS (0.1 mmol), using 15 mL/min flow at 70 C. A semi-automated in house assembled apparatus,[2] comprising of M50 CP3-8182-F2 pump (Vici, Valco Instruments Company Incorporated, Houston, TX, USA), 10-port VL,10POS,1/4-28 valve (Vici, Valco Instruments Company Incorporated, Houston, TX, USA), custom-made flow heater, equipped with plunger and empty 10 g fritted column (Biotage, Uppsala, Sweden), as a reaction vessel, was used.
The swelling of Fmoc-Rink amide-TentaGel S NH_2 90 um aminomethyl resin was performed using DMF (180 sec), prior the synthesis.
Nα-Fmoc protecting group removal was performed using 20% piperidine in DMF, containing 5% formic acid (40 sec), followed by the wash with DMF (55 sec).
For the coupling of amino acids, a mixture of Fmoc-AA-OH (10.0 equiv., 0.4 M), HCTU (9.5 equiv., 0.5 M) and NMM (20.0 equiv., neat) in DMF was delivered to the resin by flow over 18 sec. For the coupling of histidine, a mixture of Fmoc-His(Trt)-OH (10.0 equiv., 0.4 M), DIC (10 equiv., neat) and 6-Cl-HOBt (10.0 equiv., 0.5 M) in DMF was delivered to the resin by flow over 18 sec. After Fmoc-AA-OH coupling, the peptidyl-resin was washed with DMF (8 sec).

2.1.3 General procedure for Split and Pool SPPS

Pilot 5-mer library and combinatorial libraries 1-4 were synthesized using Split and Pool Fmoc/tBu SPPS.
For the Pool step (0.4 mmol), a fritted glass reaction vessel was used and swelling of Fmoc-Rink amide-TentaGel S NH_2 90 um aminomethyl resin performed using DMF (5 mL), for 30 min, prior the synthesis.
Nα-Fmoc protecting group removal was performed using 20% piperidine in DMF, containing 5% formic acid (v/v, 6 mL) for 10 min, followed by the second deprotection step using fresh reagents (10 min). The peptidyl- resin was then washed with DMF (5 × 8 mL).
For the coupling of amino acids, a mixture of Fmoc-AA-OH (5.0 equiv., 2.0 mmol), HCTU (4.75 equiv., 1.9 mmol) and NMM (10.0 equiv., 4.0 mmol) in DMF (4 mL) was added to the resin and reaction vessel shaken for 30 min. The peptidyl resin was then drained and washed with DMF (3 x 8 mL).

---

[2] A semi-automated flow SPPS apparatus was assembled in house, as a part of Maurice Wilkins Centre for Molecular Biodiscovery Flagship 3 Fund, Personalised Therapeutic Vaccines, R. Kowalczyk and P.W.R. Harris).

Acetylation of unreacted Nα-amino groups, after the Split step, was performed using 20% acetic anhydride in DMF (v/v, 5 mL) and iPr2NEt (10.0 equiv., 4.0 mmol) for 10 min, followed by the second acetylation step using fresh reagents (10 min). The peptidyl- resin was then washed with DMF (5 × 8 mL).
Before Split step, the peptidyl resin (0.4 mmol) was transferred to a 50 mL centrifuge tube. This was followed by addition of DMF (15 mL). The suspension was centrifugated (2 min, 7000x g) (Eppendorf MiniSpin Plus #5453), supernatant removed and peptidyl-resin evenly distributed into 'n' 1.5 mL tubes, corresponding to the 'n' Fmoc-AA-OH options for coupling at each position.

For the $_2$ Split step (peptide-library dependent, variable peptide synthetic scale), 1.5 mL tubes were used:
After the Pool step, each of the 'n' 1.5 mL tubes containing peptidyl resin was centrifugated (2 min, 7000x g), and supernatant removed.
For the coupling of amino acids, a mixture of the corresponding Fmoc-AA-OH (5.0 equiv.), HCTU (4.75 equiv.) and NMM (10.0 equiv.) in DMF (at the volume to reach 1 M final concentration of the Fmoc-AA-OH) was added to the respective 'n' 1.5 mL tube with peptidyl-resin and shaken for 30 min, followed by centrifugation (2 min, 7000x g), and removal of the supernatant. DMF (0.5 mL) was then added to each of the 'n' 1.5 mL tubes, peptidyl resin shaken for 3 min, and the supernatant removed. This step was repeated 3 times, after which each 'n' 1.5 mL tube containing peptidyl resin was filled with DMF (0.5 mL). The content of each of the 'n' 1.5 mL tubes was then transferred to the fritted glass reaction vessel, for subsequent Pool steps.

2.2 Design of combinatorial libraries

2.2.1 Pilot 5-mer library

Pilot 5-mer library was designed with general sequence: **I-Y-K**-X-**G-**NH_2; where Fmoc-Ile-OH (I), Fmoc-Tyr (tbu)-OH (Y), Fmoc-Lys (Boc)-OH (K), Fmoc-Gly-OH (G) amino acids are fixed while the X position is variable. The 'X' position was coupled with varied amino acids such as Fmoc-Glu(OtBu)-OH, Fmoc-Arg(Pdf)-OH, Fmoc-His(Trt)-OH, Fmoc-Leu-OH, Fmoc-Val-OH, Fmoc-Ser(tBu)-OH, Fmoc-Trp(Boc)-OH, Fmoc-Gln(Trt)-OH, Fmoc-Phe-OH.

2.2.2 Binary alanine scanning 256 member library or Library 2

This library was designed with the general sequence: X_1–X_2–X_3–X_4–X_5–X_6–X_7–X_8–**K-NH_2**, where each position consists of a binary mixture of amino acid or alanine residues except C-terminal Fmoc-Lys (Boc)-OH (K) which is fixed. X_1 is Fmoc-Ser(tBu)-OH (S) or Fmoc-Ala-OH (A); X_2: Fmoc-Asp(OtBu)-OH (D) or Fmoc-Ala-OH (A); X_3: Fmoc-Gln(Trt)-OH (Q) or Fmoc-Ala-OH (A); X_4: Fmoc-Ile-OH (I) or Fmoc-Ala-OH (A); X_5: Fmoc-Val-OH (V) or Fmoc-Ala-OH (A); X_6: Fmoc-Pro-OH (P) or Fmoc-Ala-OH (A); X_7: Fmoc-Glu(OtBu)-OH (E) or Fmoc-Ala-OH (A); X_8: Fmoc-Tyr (tBu)-OH (Y) or Fmoc-Ala-OH (A)

2.2.3 VP focused canonical amino acid 262144-member library or Library 3

This library was designed with the general sequence: X_1–X_2–X_1–**V**–**P**–X_2–X_1–X_2–**K-NH_2**, where the Fmoc-Val-OH and Fmoc-Pro-OH (VP motif) and C-terminal Fmoc-Lys (Boc)-OH (K) are fixed positions, and combinatorial diversity is introduced at defined variable positions X_1 and X_2 . X_1 position was coupled with Fmoc-Asp(OtBu)-OH (D), Fmoc-Phe-OH (F),

Fmoc-His(Trt)-OH (H), Fmoc-Lys (Boc)-OH (K), Fmoc-Leu-OH (L), Fmoc-Met-OH (M), Fmoc-Pro-OH (P), Fmoc-Trp(Boc)-OH (W); X_2 (natural amino acids): Fmoc-Ala-OH (A), Fmoc-Glu(OtBu)-OH (E), Fmoc-Gly-OH (G), Fmoc-Gln(Trt)-OH (Q), Fmoc-Ser(tBu)-OH (S), Fmoc-Thr(tBu)-OH (T), Fmoc-Val-OH (V), Fmoc-Tyr (tbu)-OH (Y)

2.2.4 VP focused non-canonical amino acid 262144-member library or Library 4

This library was designed with the general sequence: X_2–X_1–X_2–**V**–**P**–X_1–X_2–X_1–**K-NH_2**, VP motif and C-terminal Fmoc-Lys (Boc)-OH (K) are fixed positions, and the combinatorial diversity arises from defined sets of natural and non-natural amino acids.X_1 (natural amino acids): Fmoc-Val-OH (V), Fmoc-Leu-OH (L), Fmoc-Asp(OtBu)-OH (D), Fmoc-Gln(Trt)-OH (Q), Fmoc-Lys (Boc)-OH (K), Fmoc-Glu(OtBu)-OH (E), Fmoc-Met-OH (M), Fmoc-His(Trt)-OH (H); **X_2** (non-natural amino acids): Fmoc-D-Ala-OH, Fmoc-1-amino-1-cyclopropanecarboxylic acid, Fmoc-L-propargylglycine, Fmoc-D-Dab(Boc)-OH, Fmoc-L-β-tert-butylalanine, Fmoc-D-Cit-OH, Fmoc-D-3-(2-naphthyl)-alanine, Fmoc-Lys(Z)-OH

2.3 Crude Library Recovery and Yield Analysis

2.3.1 Mass Recovery of Library 2-4

The crude peptide obtained following cleavage and workup for Libraries 2, 3, and 4 was 41.16 mg, 264.76 mg, and 330.43 mg, respectively. The theoretical yield for each library was estimated using the average molecular weight of the peptide mixture, calculated from the highest and lowest molecular weight peptides present. For Libraries 3 and 4, the calculation also accounted for the potential formation of TFA adducts. Based on these theoretical yields, the overall crude synthesis yields for Libraries 2, 3, and 4 were determined to be 45.95%, 50.6%, and 50.2%, respectively.

2.4 Desalting and Fractionation of Libraries

The libraries (1-4) were desalted prior to their LC-MS/MS analysis using the protocol specified in Pierce High pH Reversed-Phase Peptide Fractionation Kit (Thermo Scientific #84868) prior to their analysis by LC-MS/MS (ZenoTOF 7600 Quadrupole-Time-of-Flight mass spectrometer (AB SCIEX)).

3. Characterisation of Synthesised Peptides

3.1 SDQIVPEYK (Trib1-Lys)-NH_2 (1)

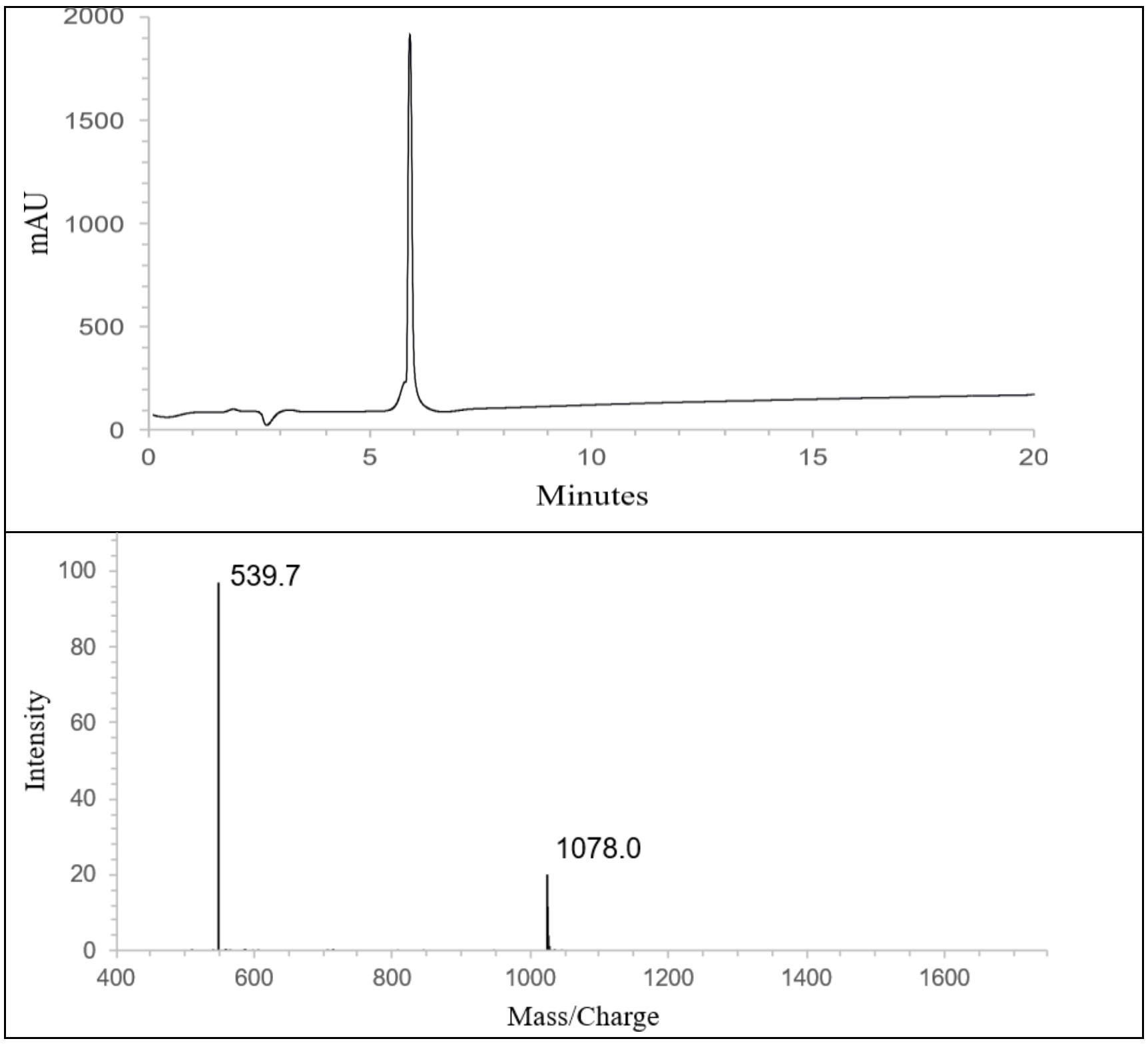

Supplementary Figure 1 LCMS traces of pure peptide SDQIVPEYK (Trib1-Lys) (**1**) (ca 97% as judged by peak area at 214 nm). Rt 7.01 min; m/z 539.7 $[M + 2H]^{2+}$ requires 539.6, m/z 1078.0 [M + H]+ requires 1078.2. Mass deconvolution calculated at 1077.20 Da with standard deviation of 0.28; theoretical mass calculated at 1077.20 Da.

3.2 5(6)-FAM-Ahx-SDQIVPAYK-NH_2 (5(6)-FAM-TRIB1-Lys) (2)

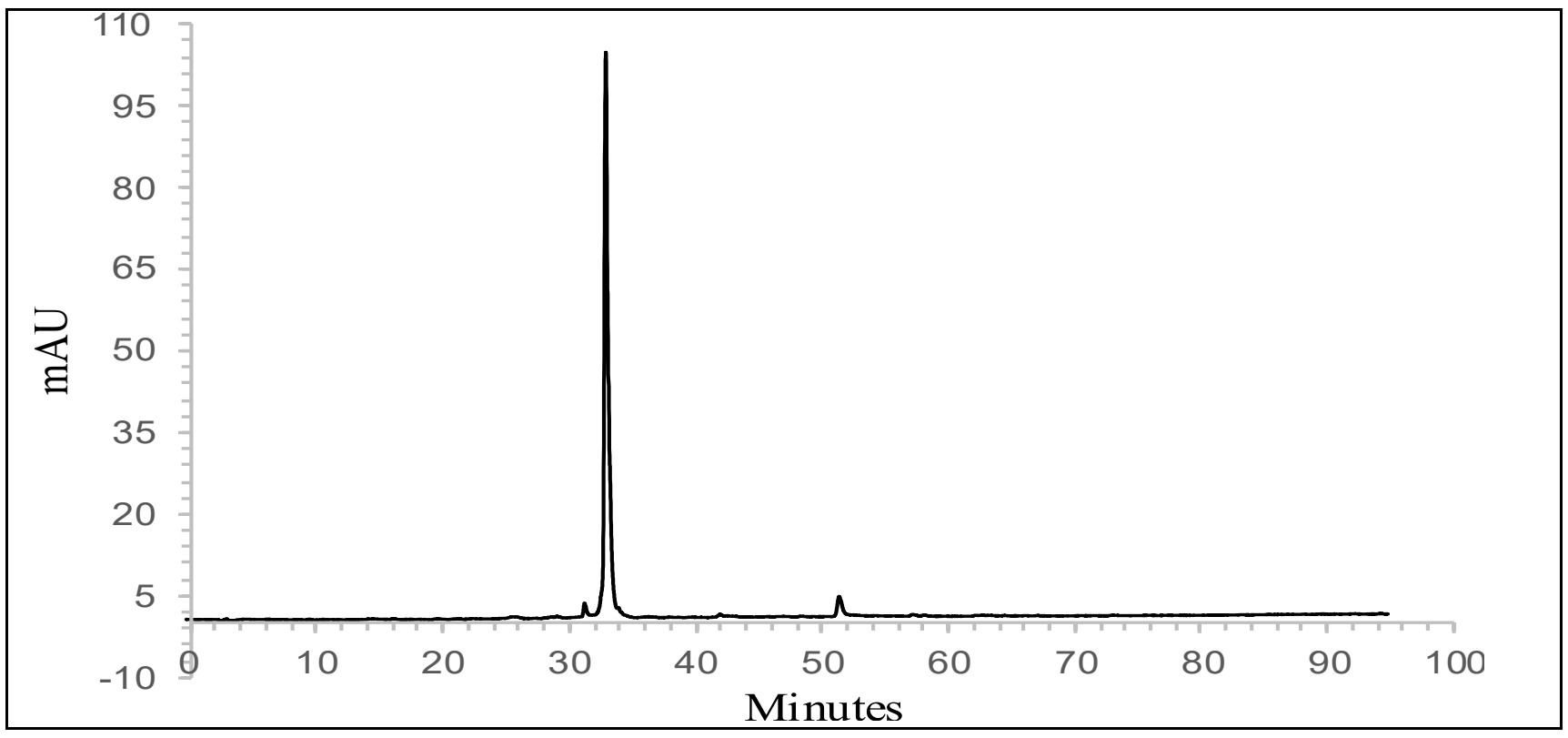

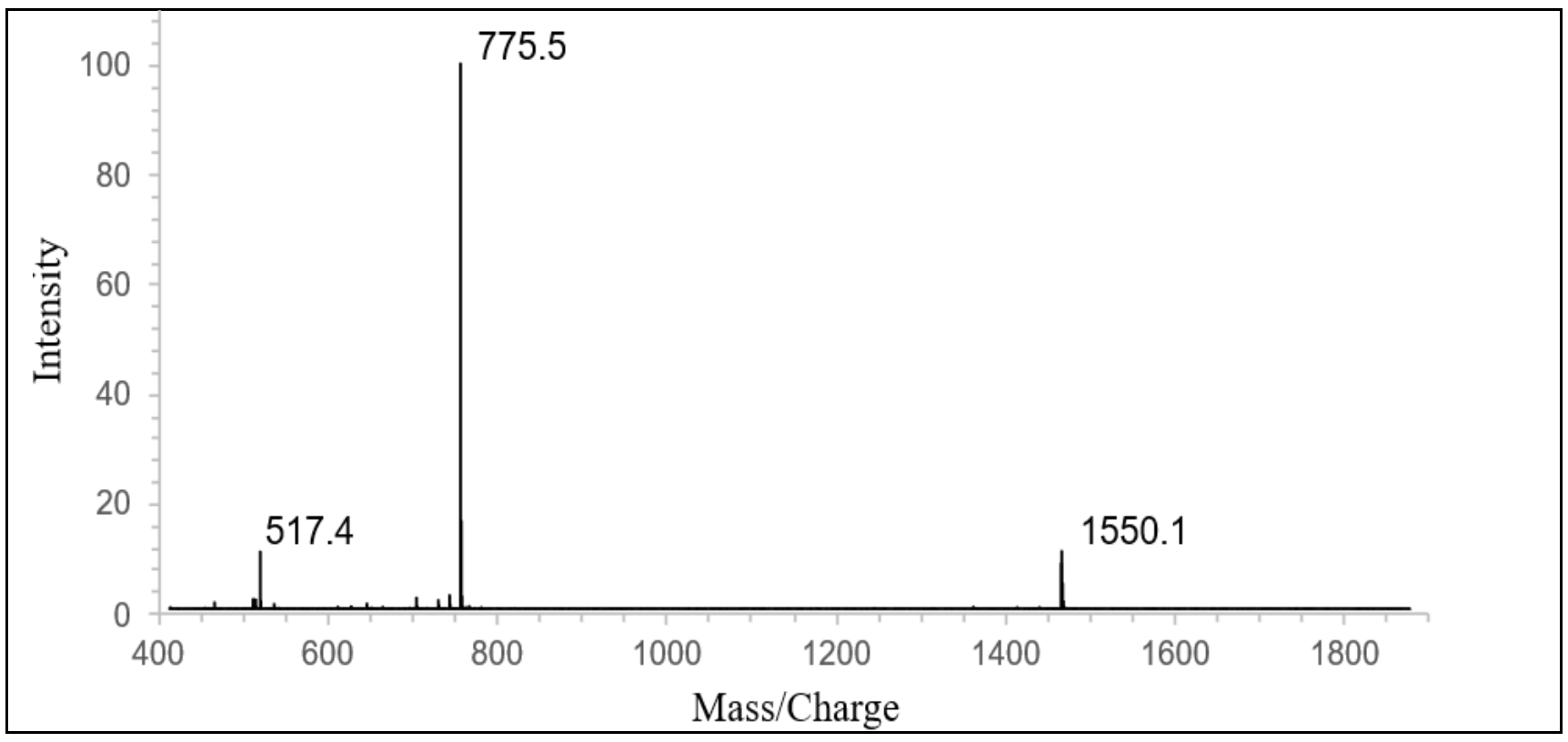

Supplementary Figure 2 RP-HPLC and ESI-MS traces of pure peptide 5(6)-FAM-Ahx-SDQIVPAYK (**2**) (ca 98% as judged by peak area of RP-HPLC at 280 nm). Rt 31.2 min; m/z 517.4 $[M + 3H]^{3+}$ requires 516.89, m/z 775.5 $[M + 2H]^{2+}$ requires 774.85, m/z 1550.1 [M + H]+ requires 1548.69. Mass deconvolution calculated at 1549.10 Da with standard deviation of 0.10; theoretical mass calculated at 1548.40 Da.

3.3 SDQIVPAYK -NH_2 (3)

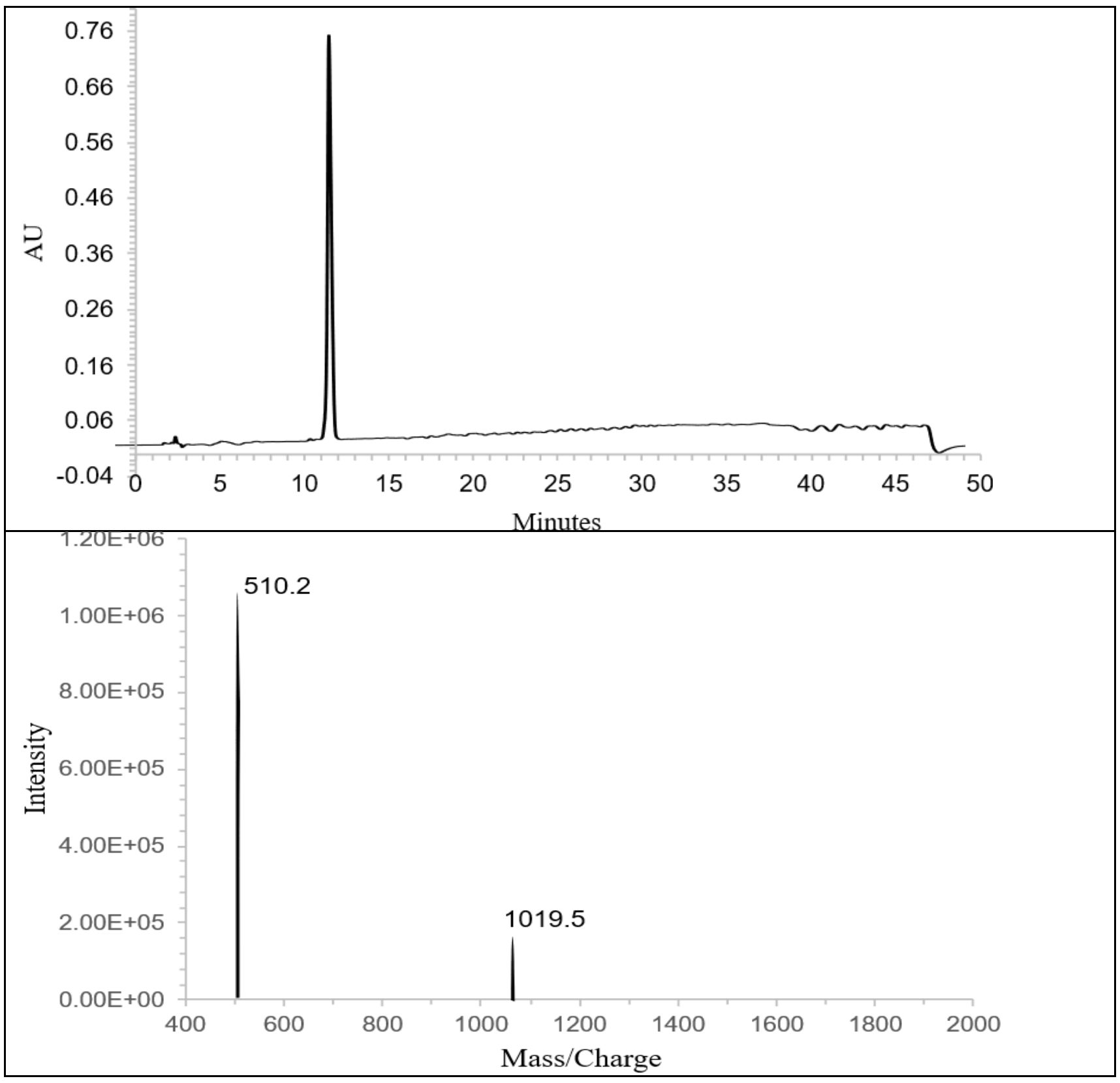

Supplementary Figure 3 RP-HPLC and HRMS traces of pure peptide SDQIVPAYK (**3**) (ca 99% as judged by peak area of RP-HPLC at 214 nm). Rt 12.84 min; m/z 510.2 $[M + 2H]^{2+}$ requires 510.58, m/z 1019.5 [M + H] + requires 1020.17. Mass deconvolution calculated at 1018.45 Da with standard deviation of 0.07; theoretical mass calculated at 1019.16 Da.

3.4 SAQIVPEYK-NH$_2$ (4)

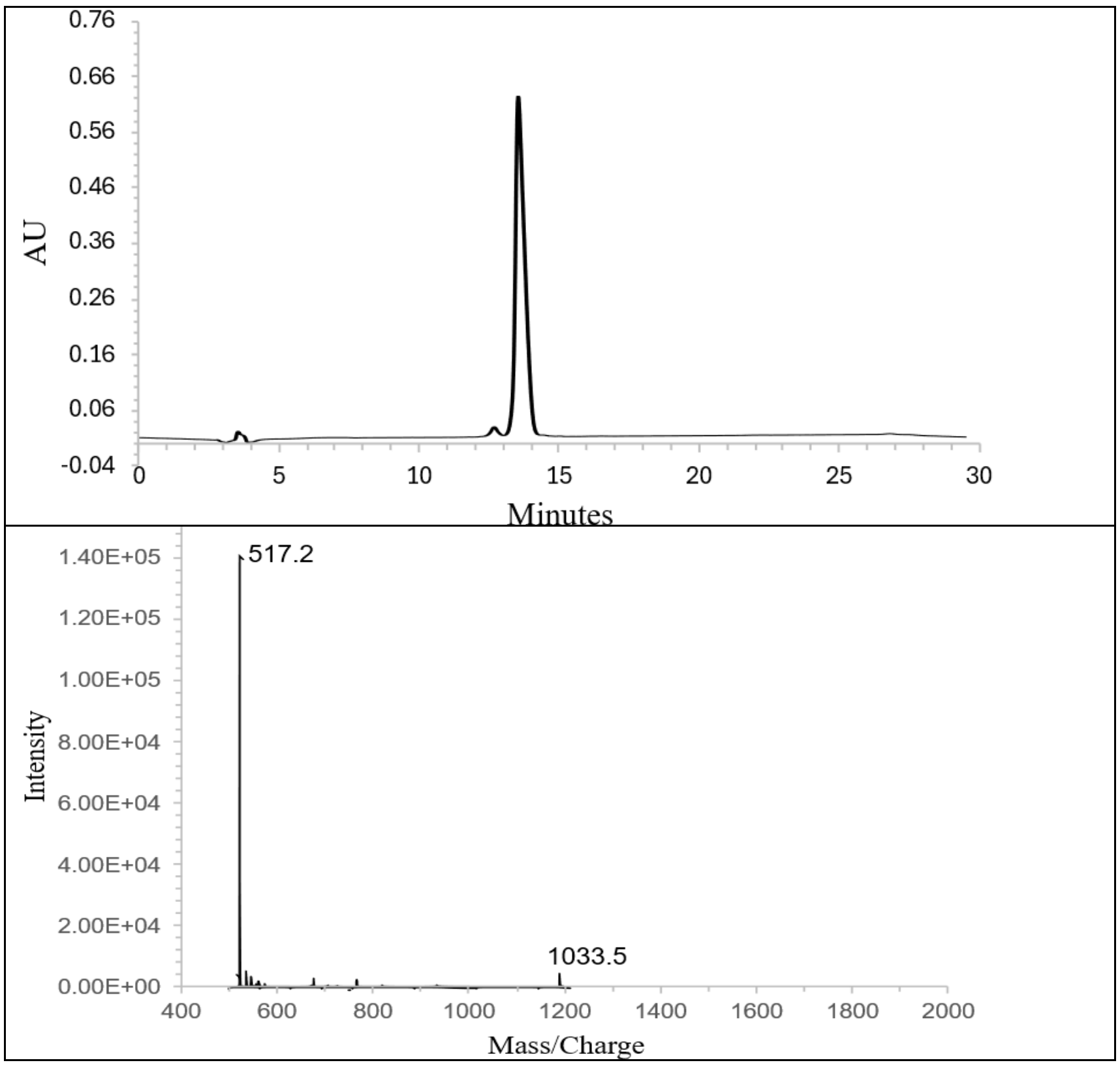

Supplementary Figure 4 RP-HPLC and HRMS traces of pure peptide SAQIVPEYK (**4**) (ca 98% as judged by peak area of RP-HPLC at 214 nm). Rt 13.12 min; m/z 517.2 $[M + 2H]^{2+}$ requires 517.60, m/z 1033.5 [M + H] + requires 1034.19. Mass deconvolution calculated at 1032.45 Da with standard deviation of 0.07; theoretical mass calculated at 1033.18 Da.

3.5 SAQIVPAYK-NH$_2$ (5)

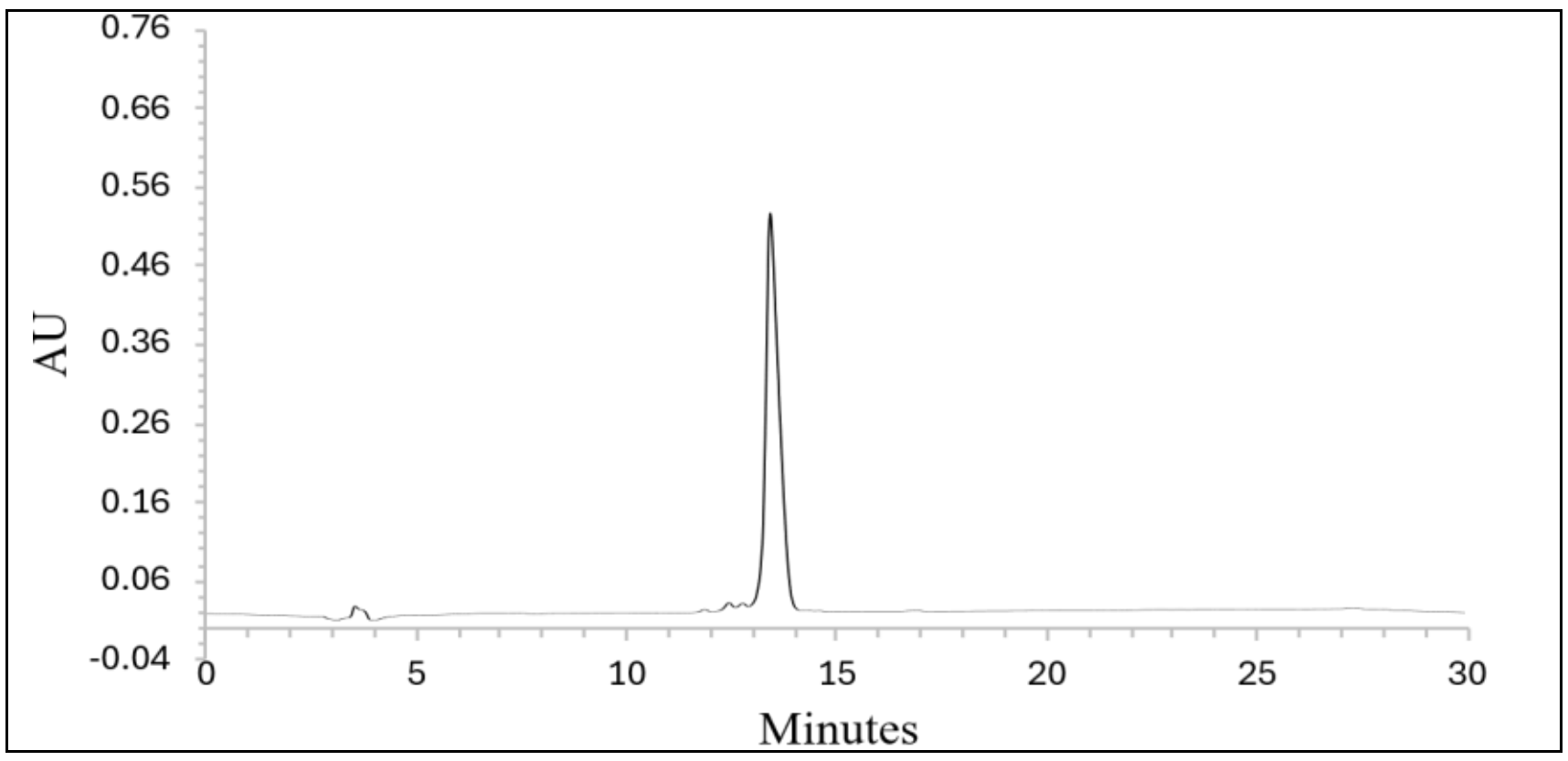

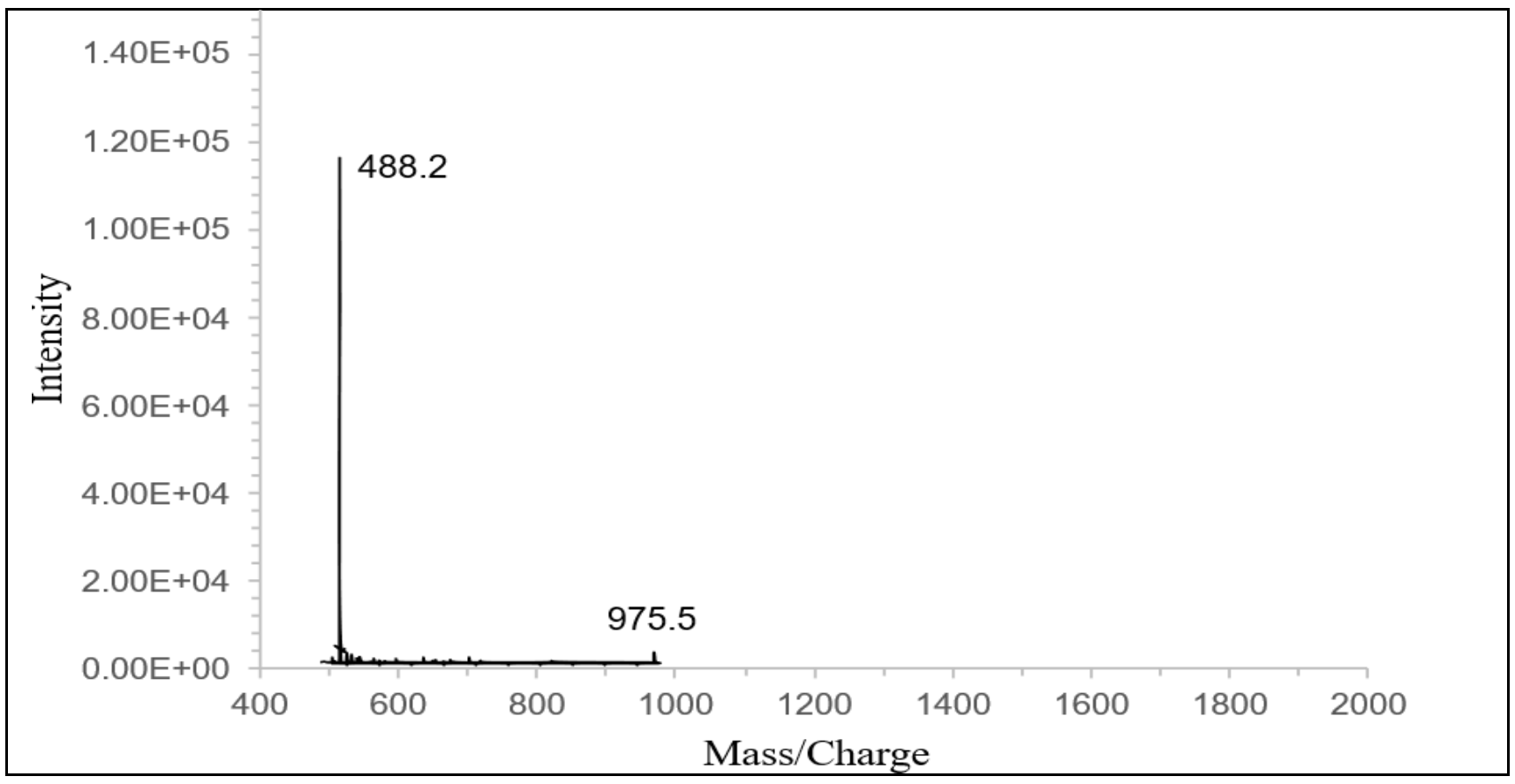

Supplementary Figure 5 RP-HPLC and HRMS traces of pure peptide SAQIVPAYK (**5**) (ca 99% as judged by peak area of RP-HPLC at 214 nm). Rt 12.87 min; m/z 488.2 $[M + 2H]^{2+}$ requires 494.62, m/z 975.5 [M + H] + requires 988.23. Mass deconvolution calculated at 974.45 Da with standard deviation of 0.07; theoretical mass calculated at 987.22 Da.

3.6 TDQLVPVWK-NH_2 (6)

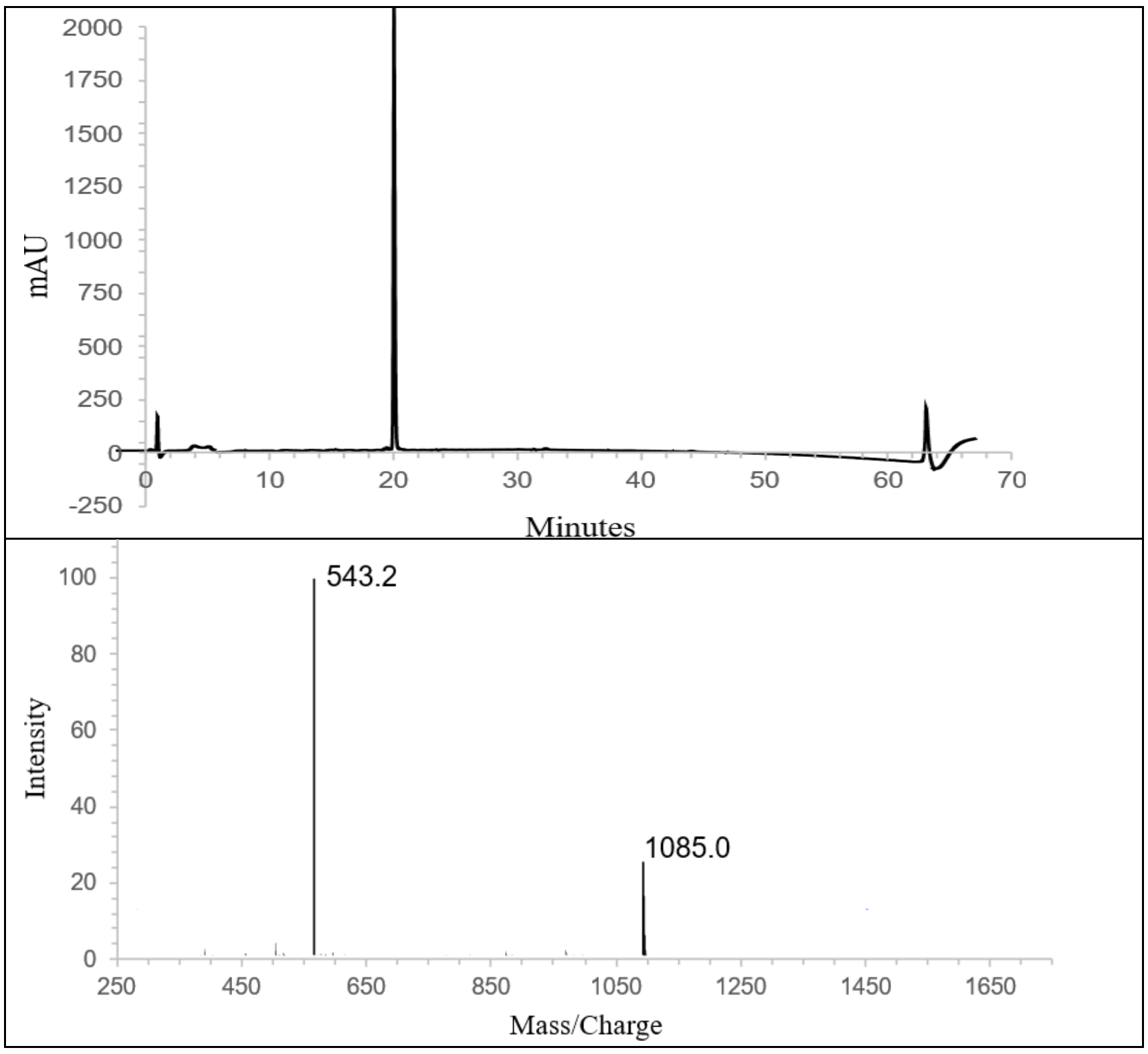

Supplementary Figure 6 RP-HPLC and ESI-MS traces of pure peptide TDQLVPVWK (**6**) (ca 99% as judged by peak area of RP-HPLC at 214 nm). Rt 19.95 min; m/z 543.2 $[M + 2H]^{2+}$ requires 543.14, m/z 1085.0 [M + H]+ requires 1085.28. Mass deconvolution calculated at 1084.20 Da with standard deviation of 0.28; theoretical mass calculated at 1084.28 Da.

3.7 VLALYLTPK-NH_2 (7)

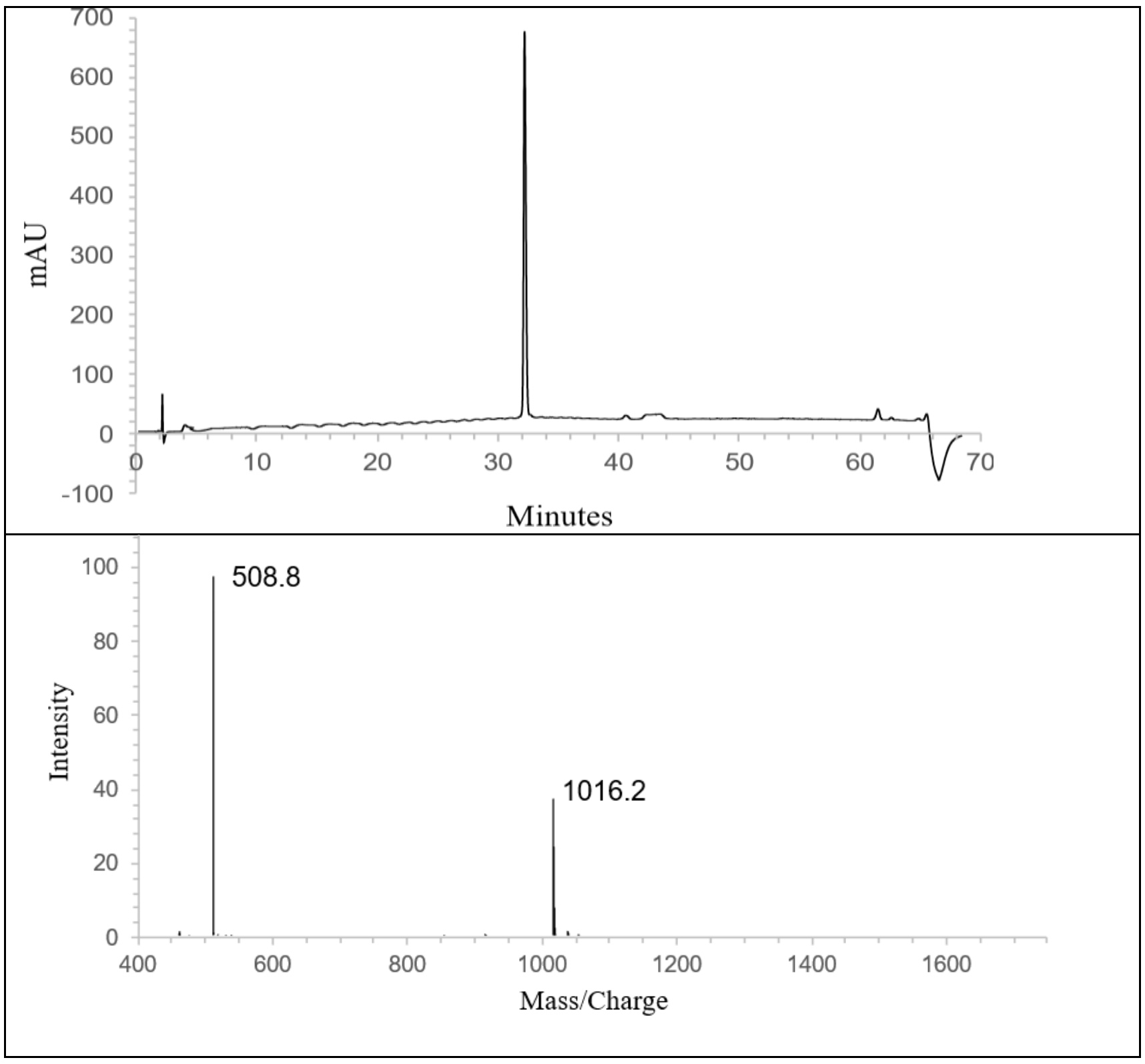

Supplementary Figure 7 RP-HPLC and ESI-MS traces of pure peptide VLALYLTPK (**7**) (ca 99% as judged by peak area of RP-HPLC at 214 nm). Rt 31.99 min; m/z 508.8 $[M + 2H]^{2+}$ requires 509.15, m/z 1016.2 [M + H] + requires 1017.29. Mass deconvolution calculated at 1015.40 Da with standard deviation of 0.28; theoretical mass calculated at 1016.28 Da.

3.8 SFALVPVLK-NH_2 (8)

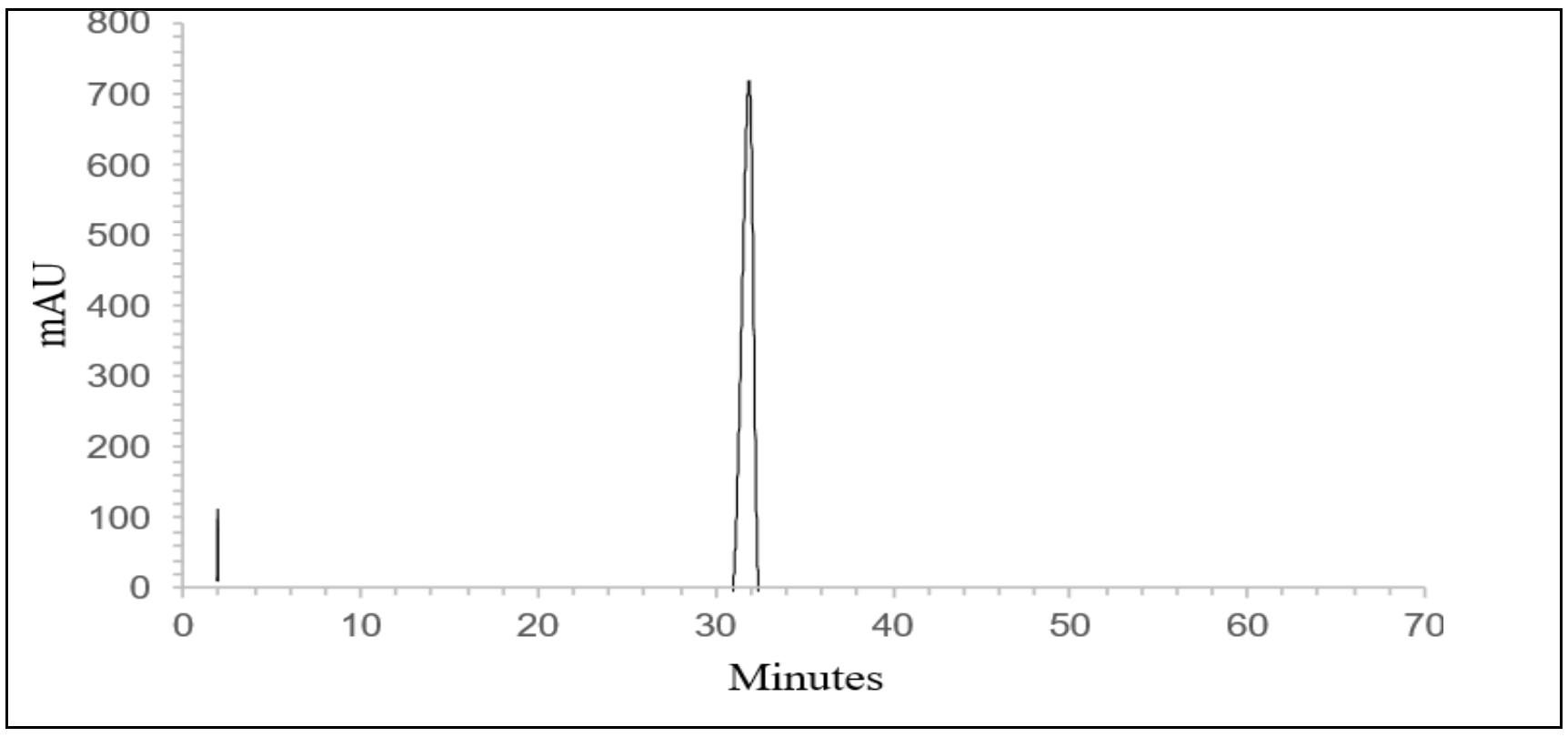

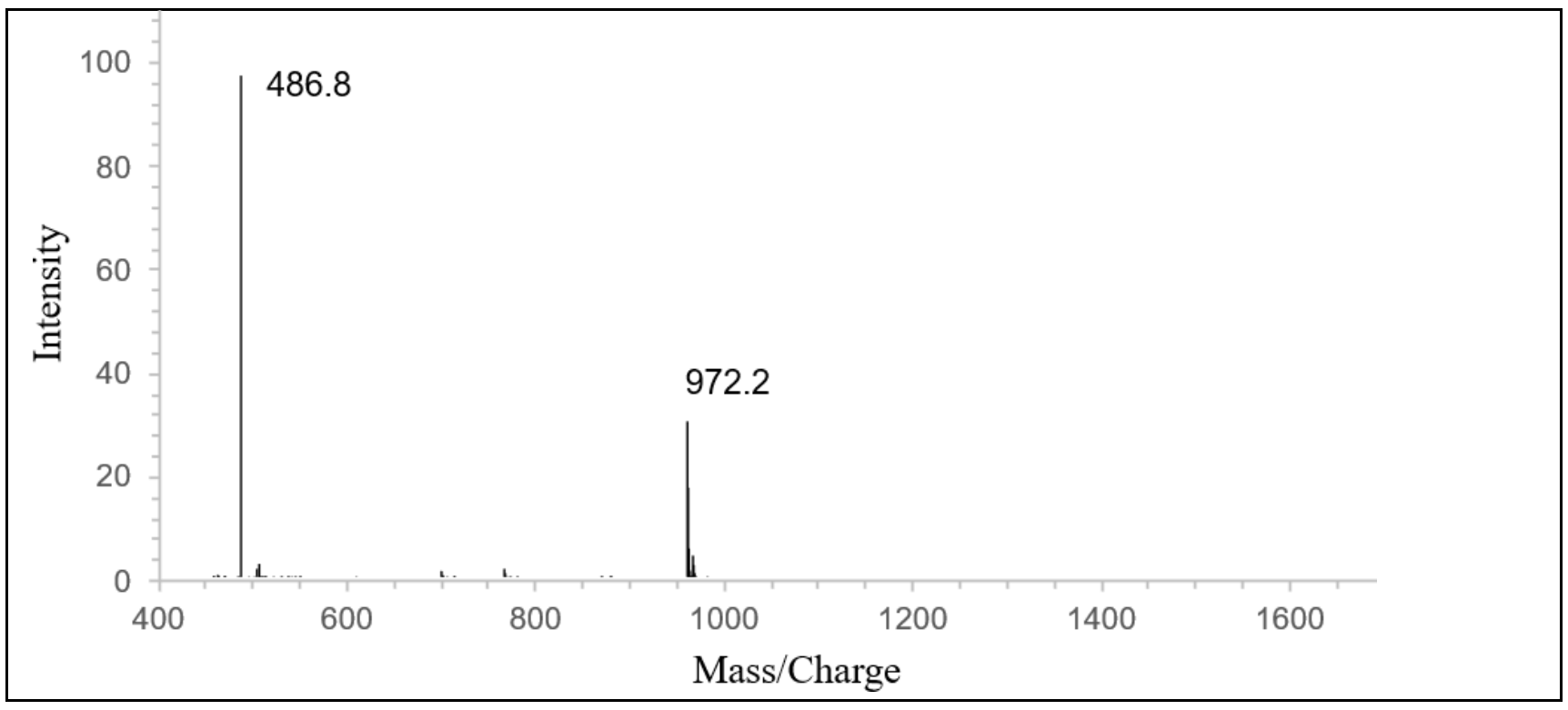

Supplementary Figure 8 RP-HPLC and ESI-MS traces of pure peptide SFALVPVLK (**8**) (ca 98% as judged by peak area of RP-HPLC at 214 nm). Rt 30.6 min; m/z 486.8 $[M + 2H]^{2+}$ requires 487.1, m/z 972.2 [M + H]+ requires 973.2. Mass deconvolution calculated at 971.4 Da with standard deviation of 0.28; theoretical mass calculated at 972.20 Da.

3.9 TFALVPGFK-NH_2 (9)

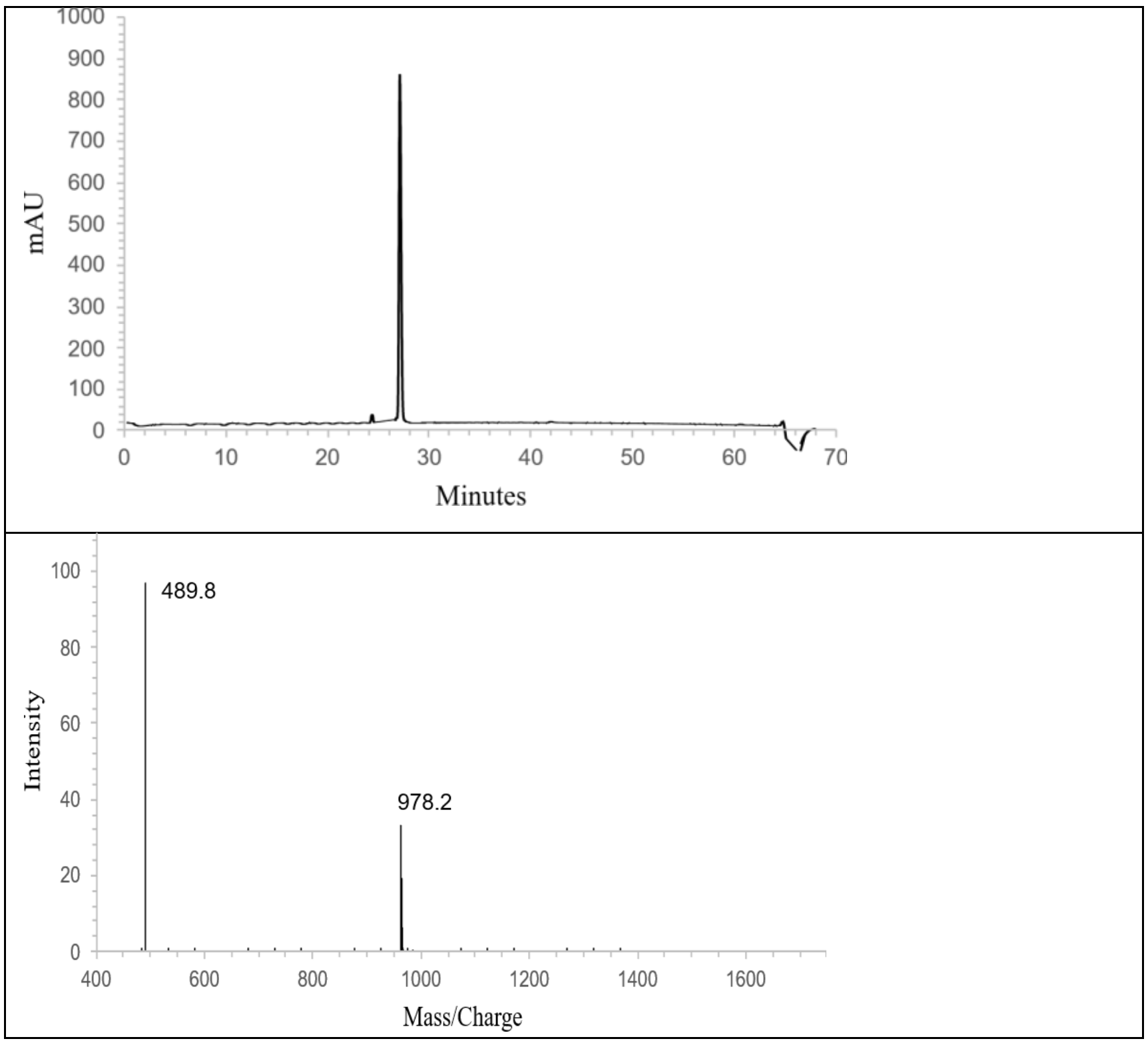

Supplementary Figure 9 RP-HPLC and ESI-MS traces of pure peptide TFALVPGFK (**9**)) (ca 95% as judged by peak area of RP-HPLC at 214 nm). Rt 29.30 min; m/z 489.8 $[M + 2H]^{2+}$ requires 490.11, m/z 978.2 [M + H] + requires 979.21. Mass deconvolution calculated at 977.40 Da with standard deviation of 0.28; theoretical mass calculated at 978.19 Da.

3.10 QLVWVFVPK-NH_2 (10)

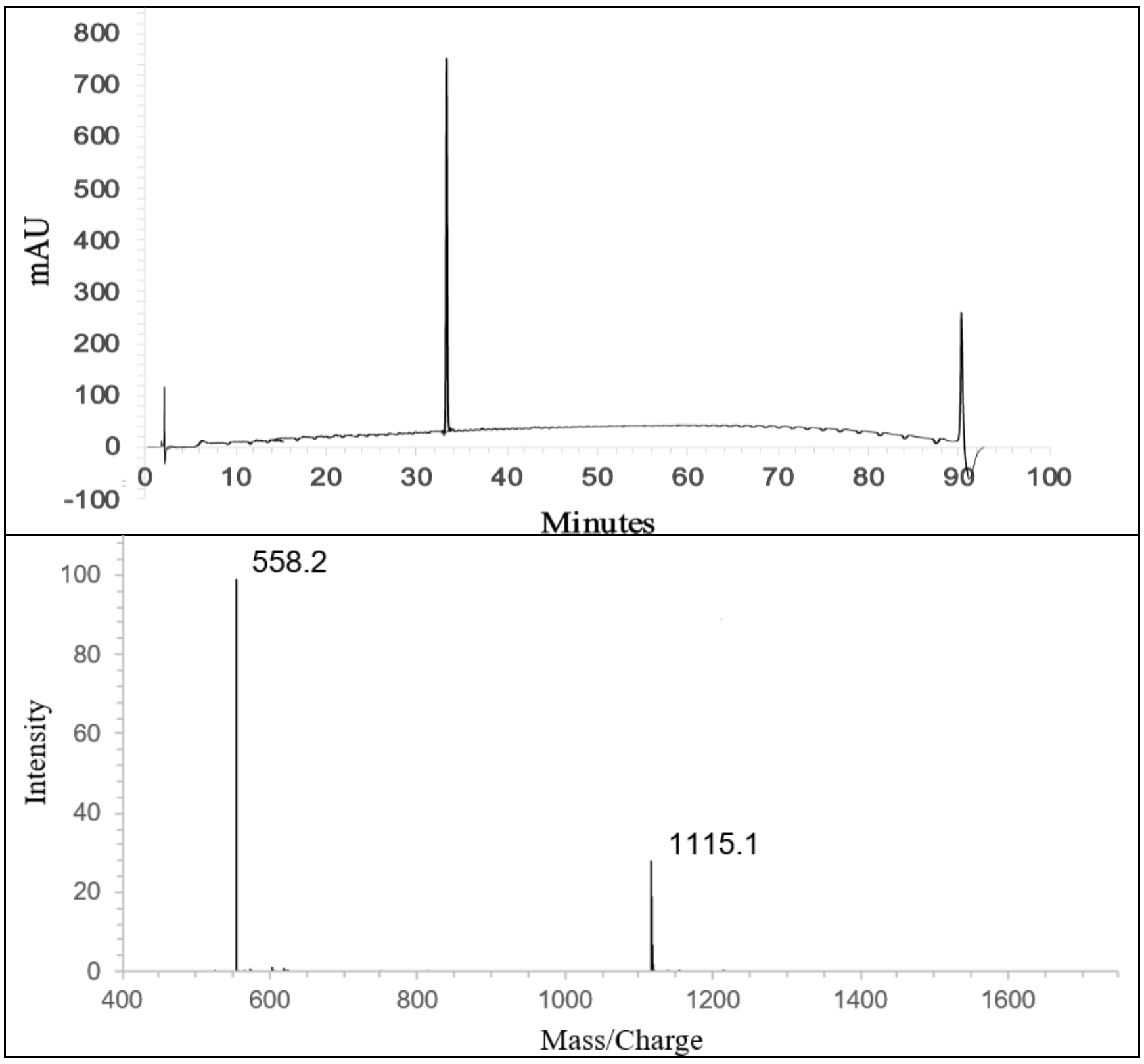

Supplementary Figure 10 RP-HPLC and ESI-MS traces of pure peptide QLVWVFVPK **10**) (ca 95% as judged by peak area of RP-HPLC at 214 nm). Rt 29.30 min; m/z 558.2 $[M + 2H]^{2+}$ requires 558.21, m/z 1115.1 [M + H] + requires 1115.40. Mass deconvolution calculated at 1114.25 Da with standard deviation of 0.21; theoretical mass calculated at 1114.3954 Da.

3.11 KTDVPAMVK-NH$_2$ (11)

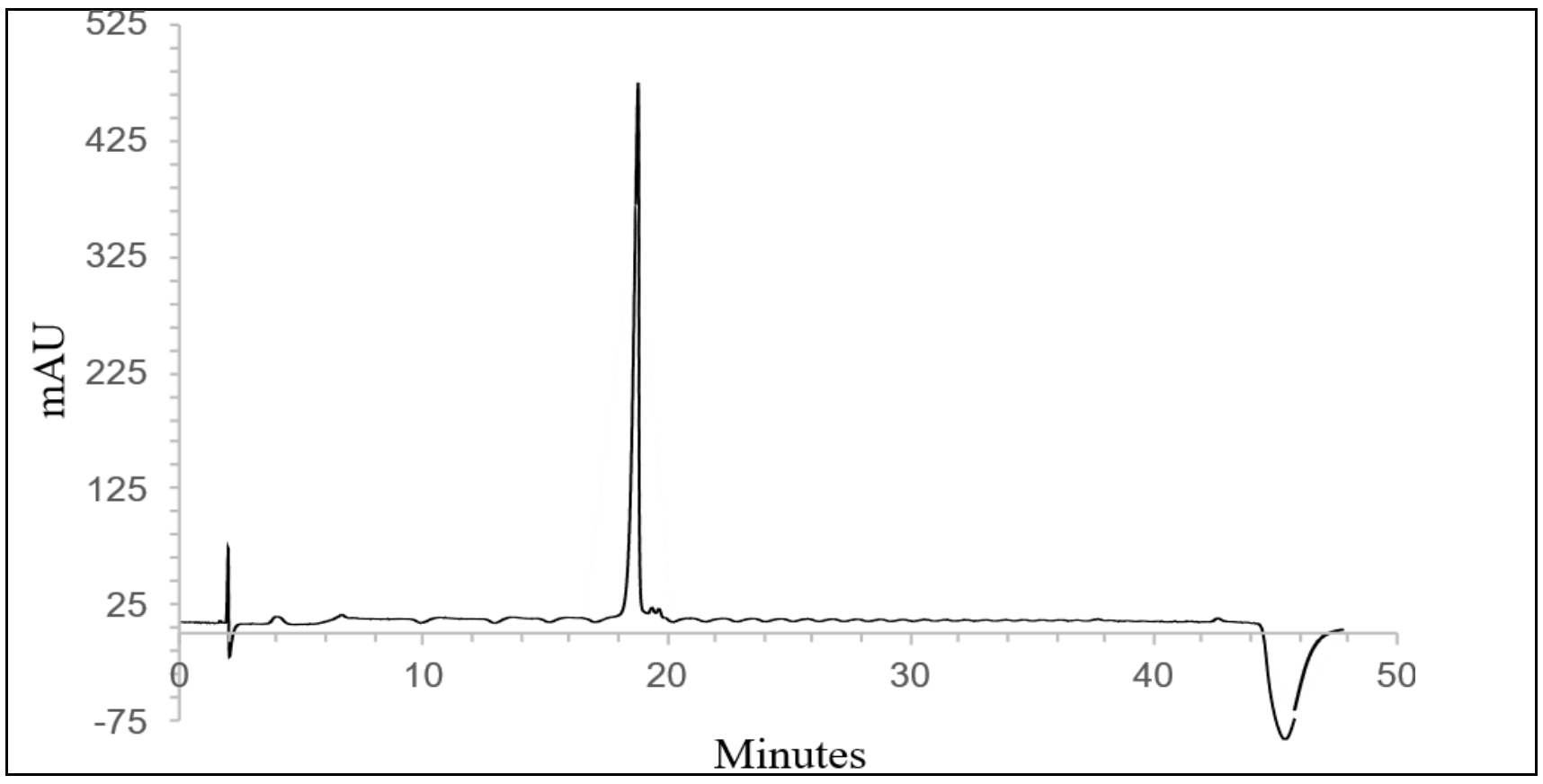

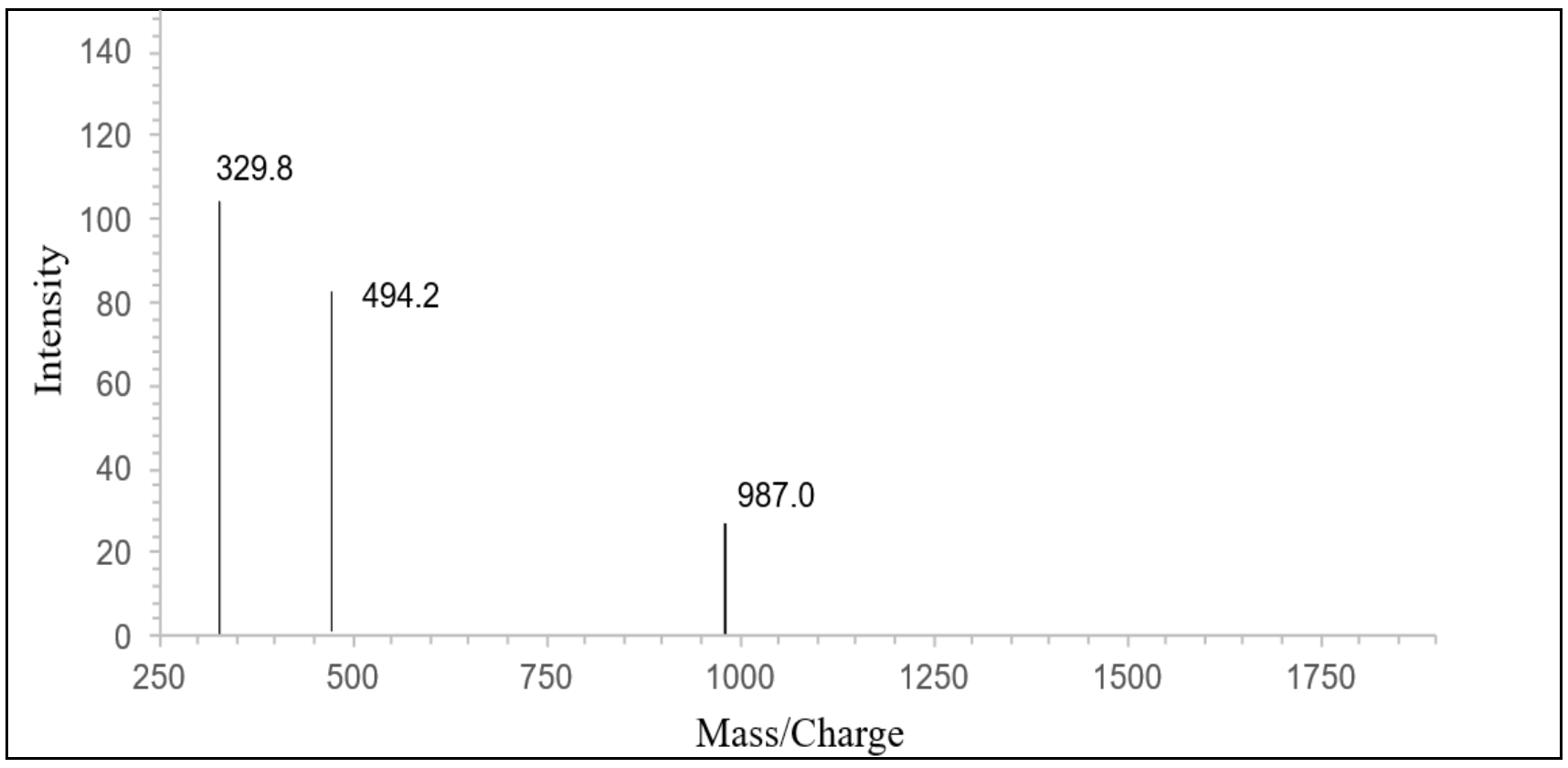

Supplementary Figure 11 RP-HPLC and ESI-MS traces of pure peptide KTDVPAMVK (**11**) (ca 96% as judged by peak area of RP-HPLC at 214 nm). Rt 18.85 min; m/z 329.8 $[M + 3H]^{3+}$ requires 330.08, m/z 494.2 $[M + 2H]^{2+}$ requires 494.62, m/z 987.0 [M + H] + requires 988.23. Mass deconvolution calculated at 986.27 Da with standard deviation of 0.23; theoretical mass calculated at 987.22 Da.

3.12 KYMVPVMSK-NH_2 (12)

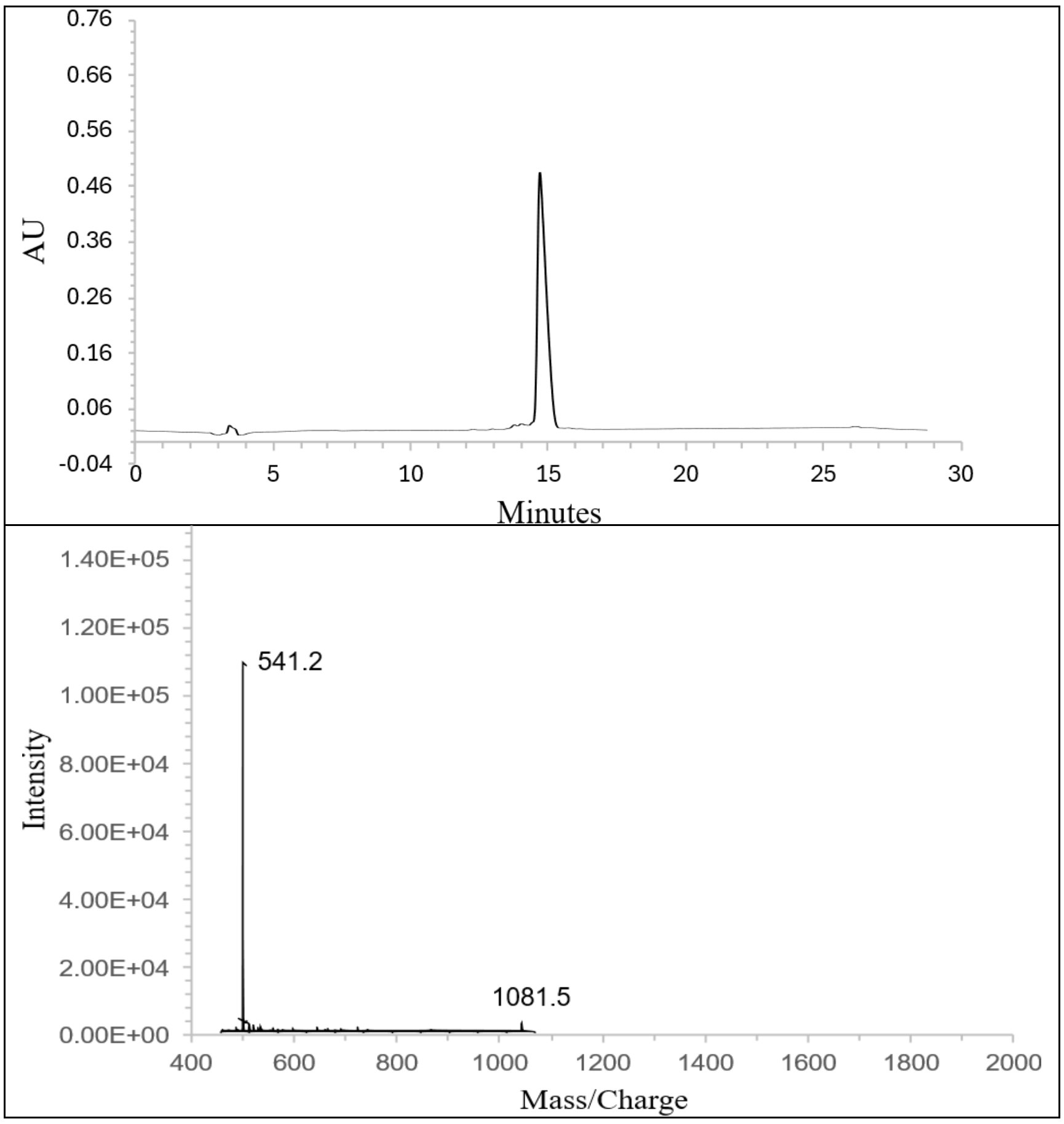

Supplementary Figure 12 RP-HPLC and HRMS traces of pure peptide KYMVPVMSK (**12**) (ca 99% as judged by peak area of RP-HPLC at 214 nm). Rt 14.69 min; m/z 541.2 $[M + 2H]^{2+}$ requires 541.71, m/z 1081.5 [M + H]+ requires 1082.40. Mass deconvolution

calculated at 1080.45 Da with standard deviation of 0.07; theoretical mass calculated at 1081.39 Da.

3.13 NEUVPVWEK-NH_2 (13)

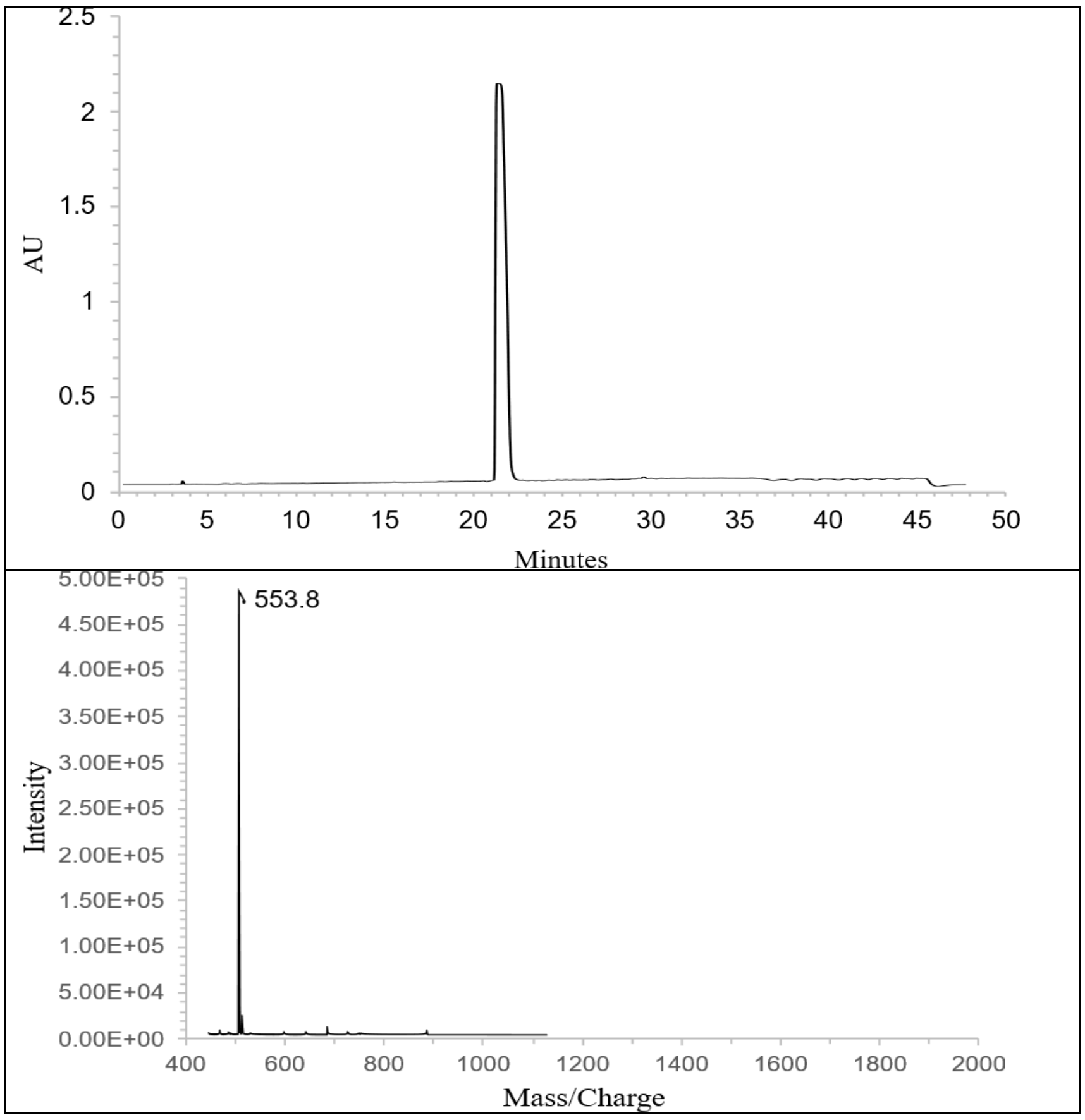

Supplementary Figure 13 RP-HPLC and HRMS traces of pure peptide NEUVPVWEK (**13**) (ca 99% as judged by peak area of RP-HPLC at 214 nm). Rt 21.78 min; m/z 553.8 $[M + 2H]^{2+}$ requires 554.68. Mass deconvolution calculated at 1105.6 Da the standard deviation could not be calculated due to only one value; theoretical mass calculated at 1107.36 Da.

3.14 KSMVPAFYK-NH_2 (14)

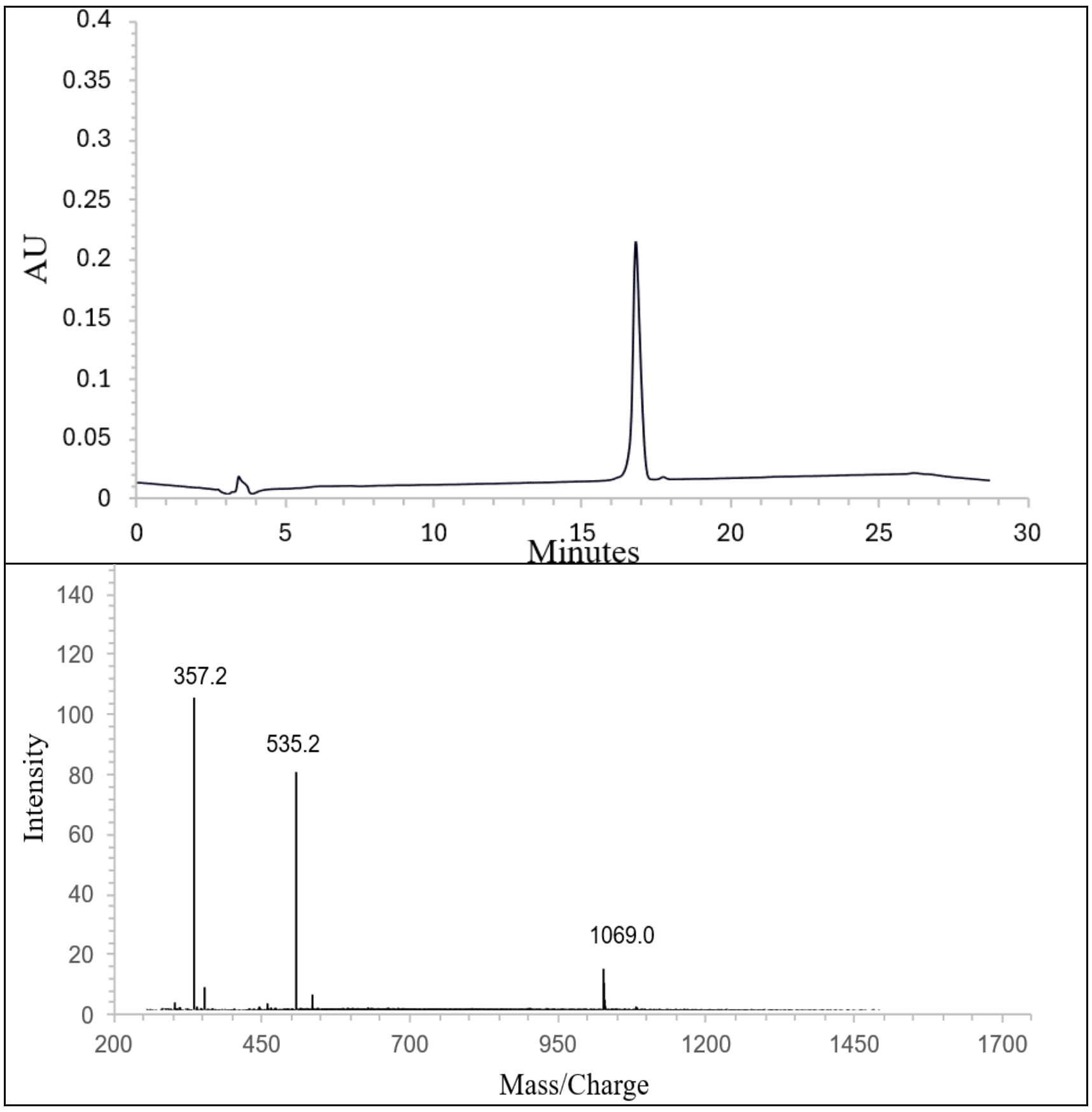

Supplementary Figure 14 RP-HPLC and HRMS traces of pure peptide KSMVPAFYK (**14**) (ca 99% as judged by peak area of RP-HPLC at 214 nm). Rt 16.38 min; m/z 357.2 [M + 3H]3+ requires 357.45, m/z 535.2 $[M + 2H]^{2+}$ requires 535.67, m/z 1069.0 [M + H]+ requires 1070.33. Mass deconvolution calculated at 1068.33 Da with standard deviation of 0.31; theoretical mass calculated at 1069.33 Da.

3.15 KGHVPQDSK-NH_2 (15)

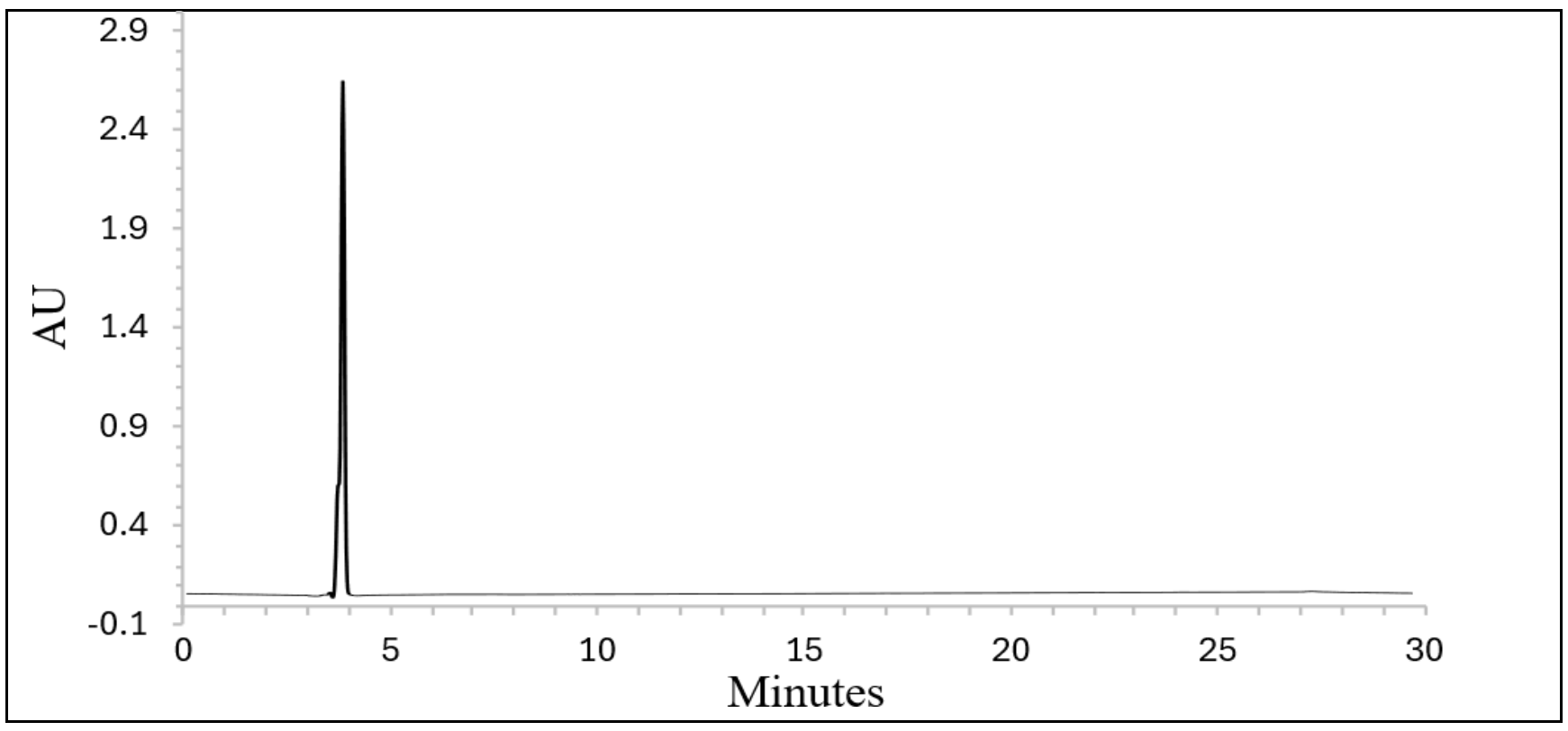

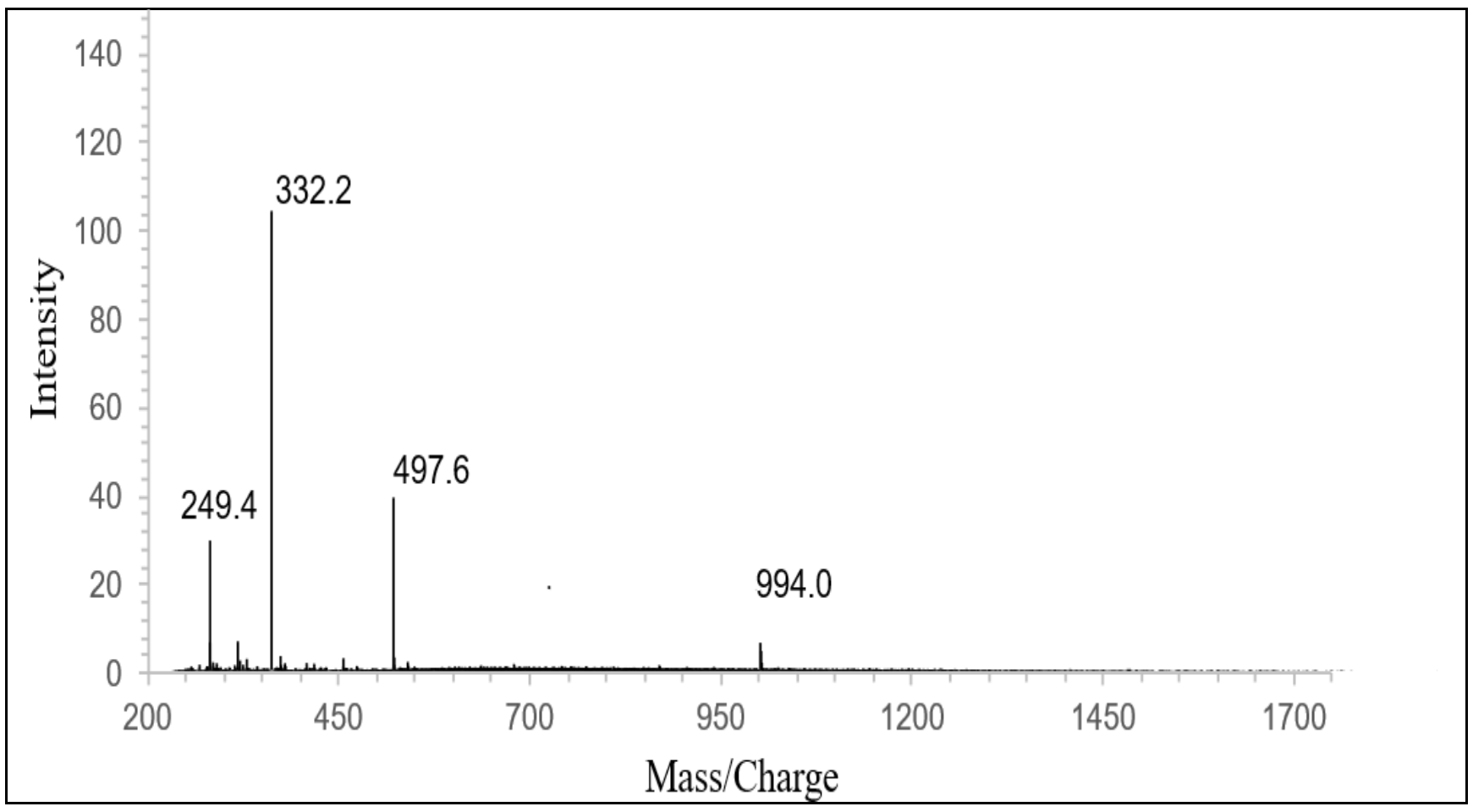

Supplementary Figure 15 RP-HPLC and HRMS traces of pure peptide KGHVPQDSK (**15**) (ca 99% as judged by peak area of RP-HPLC at 214 nm). Rt 3.83 min; m/z 249.4 $[M + 4H]^{4+}$ requires 249.54, m/z 332.2 $[M + 3H]^{3+}$ requires 332.38, m/z 497.6 $[M + 2H]^{2+}$ requires 498.06, m/z 994.0 [M + H] + requires 995.12. Mass deconvolution calculated at 993.35 Da with standard deviation of 0.30; theoretical mass calculated at 994.11 Da.

4 Development of the COP1 Affinity Selection Platform

4.1 Recombinant WD40 domain COP1 protein production

Arabidopsis thaliana COP1 (349-675) was cloned into the pFastBac expression vector to incorporate a N-terminal His10 tag affinity tag followed by a 3C protease cleavage site. To generate bacmid the pFastBac construct was transformed into DH10EMBacY cells (Geneva Biotech) and the bacmid isolated. Spodoptera frugiperda (Sf9) cells were transfected to produce baculoviral stocks which were then used for subsequent protein expression. The COP1-WD40 was produced in Trichoplusia ni (TNi). Cells were incubated with baculovirus at 27°C for 48 hours. Following incubation with baculovirus, cells were harvested by centrifugation at 1000g for 30 min. Cell pellets were resuspended in purification buffer [50 mM Tris-HCl (pH 8.0), 300 mM NaCl, 10% (v/v) glycerol, and 10% (w/v) sucrose]. Cells were lysed using sonication and the soluble fraction was initially purified by affinity chromatography using Ni2+ affinity resin [His-Select resin (Sigma-Aldrich)]. Proteins were eluted from the resin by increasing the imidazole concentration to 300 mM. The protein was further purified with size exclusion chromatography using a 10/300 S75 increase (Cytiva life science) equibrated with phosphate buffer saline pH 7.4, 5% glycerol. Purified protein was then aliquoted, snap frozen in liquid nitrogen and stored at -80°C.

4.2 Optimisation of COP1 Protein Loading

HisPur™ Ni-NTA magnetic beads (12.5 mg/mL) were washed three times with binding buffer (1× PBS, 0.05% Tween-20) and serially diluted to give 5, 2.5, 1.25, and 0.625 µL bead aliquots. Each aliquot was incubated with 68 pmol His-COP1 (2.5 µg, 2.13 mg/mL) for 45 min at 25 °C with shaking (1100 rpm) on Eppendorf ThermoMixer. Supernatants were collected following protein immobilisation and after a subsequent binding buffer wash. Beads

were then incubated in selection buffer (1× PBS, 0.05% Tween-20, 0.1 mg/mL BSA, pH 7.5) for 5 min and washed 3 × 100 μL with binding buffer. Bead-bound protein was recovered by heat elution following denaturation in 1× Bolt™ loading buffer containing 5% (v/v) 2-mercaptoethanol at 95 °C for 5 min. Supernatant and eluate fractions were analysed by SDS–PAGE using Bolt™ Bis-Tris Plus 4–12% gels with Precision Plus Protein Kaleidoscope™ standards. Gels were run at 150 V for 45 min, stained with Coomassie Brilliant Blue, destained (destaining solution containing 50% water, 40% methanol, 10% acetic acid), and imaged using an Invitrogen iBright™ 1500 system.

4.3 Optimisation of the affinity selection using 5(6)-FAM-TRIB1-Lys

A fluorescently labelled TRIB1 peptide analogue **(peptide 2)** containing an N-terminal carboxyfluorescein linked via a 6-aminohexanoic acid spacer was used to optimise COP1 affinity selection conditions. Immobilised COP1 and bead-only control samples were incubated with 5(6)-FAM-TRIB1-Lys (10 μM, 250 pmol in 25 μL selection buffer consisting of 1× PBS, 0.05% Tween-20, and 0.1 mg/mL BSA), while no-peptide controls received selection buffer alone. Samples were incubated for 1 h at 25 °C with shaking (1100 rpm), washed 3 × 200 μL with binding buffer, and bound peptide was eluted in 25 μL 1× PBS by heating at 80 °C for 10 min. Competition experiments were performed by co-incubating Peptide 2 (10 μM, 250 pmol) with non-fluorescent **peptide 1** at concentrations ranging from 4 μM to 1 mM (100 pmol–25 nmol), while concentration-response experiments evaluated **peptide 2** concentrations from 137 nM to 33 μM (3.4–825 pmol). The effects of incubation temperature and elution method were assessed by comparing selections performed at 4 °C and 25 °C, with peptide elution achieved either by heating in 1× PBS (80 °C, 10 min) or with 6 M guanidine hydrochloride in 200 mM sodium phosphate buffer (pH 6.8) for 10 min at room temperature. Fluorescence was measured using a PerkinElmer EnSpire plate reader (λ_{ex} = 500 nm, λ_{em} = 520 nm), and 5(6)-FAM-TRIB1-Lys concentrations were determined from a 12-point calibration curve (0.5 nM–100 μM) using Microsoft Excel and GraphPad Prism (version 10).

4.4 Pilot Affinity Selection Using Library 1

A pilot affinity selection was performed using Library 1 against immobilised COP1. COP1 + library, COP1 without library, and bead-only (no-protein control) conditions were prepared in technical triplicate. Library-containing samples were incubated with 25 μL of Library 1 (20 μM total library concentration; 30 fmol per library member) in selection buffer, while no-library controls received selection buffer only. Following incubation and washing as described above, bound peptides were eluted in 11 μL 1× PBS by heating at 80 °C for 10 min. Eluates were quantified using the Pierce™ Quantitative Fluorometric Peptide Assay Kit (Thermo Scientific, #23290) according to the manufacturer's instructions. Fluorescence was measured in black 96-well plates using a PerkinElmer EnSpire plate reader (λ_{ex} = 390 nm, λ_{em} = 475 nm), and peptide concentrations were determined from a six-point calibration curve (4 μM–1 mM) with PBS blanks. Data were analysed using Microsoft Excel and GraphPad Prism (version 10).

5. Affinity Selection Mass Spectrometry Screening

5.1 Affinity selection with Library 1

HisPur™ Ni-NTA magnetic beads (12.5 mg/mL) were loaded with His-COP1 (5, 10, or 20 µg; 136–544 pmol) or prepared as no-protein controls. Affinity Selection 1 was performed using Library 1 (5 mM total concentration, 7 fmol/member) in a final volume of 25 µL. Additional 10 µg His-COP1 selection conditions were supplemented with a TRIB1 peptide binder at either 1× abundance (300 pM, 7 fmol) or 1000× abundance (300 nM, 7 pmol), where 1× and 1000x corresponds to the average abundance of an individual library member. Samples were incubated with protein-immobilised beads for 1 h at 25 °C with shaking (1100 rpm). Typical final conditions were 5–10 µg His-COP1, 2.5–5.0 µL Ni-NTA magnetic beads, and 5 mM total peptide library concentration (7 fmol per member). Beads were washed 3 × 1 mL with 1× PBS, and bound peptides were eluted in 20 µL 1× PBS by heating at 80 °C for 10 min. Eluates were collected following magnetic separation and analysed by label-free LC–MS/MS.
Affinity Selection 2 was performed as described above except that increased amounts of immobilised His-COP1 were used (10 and 20 µg; 272 and 544 pmol, respectively). Samples were incubated with 25 µL Library 1 (5 mM, 7 fmol per member), washed 3 × 1 mL with 1× PBS, and eluted in 20 µL 1× PBS at 80 °C for 10 min prior to label-free LC–MS/MS analysis.

5.2 Affinity selection with Library 2

Affinity selections were performed using His-tagged COP1 (His-COP1) immobilised on HisPur™ Ni-NTA magnetic beads. Each selection contained 5 µg His-COP1 and was performed in duplicate alongside no-protein controls. Library 2 was evaluated across a concentration series of 10 nM, 100 nM, 1 µM, 10 µM, 100 µM, and 1 mM total peptide, corresponding to approximately 1 fmol–100 pmol per individual library member. Ni-NTA magnetic beads (1.25 µL, 12.5 mg/mL) were equilibrated in binding buffer (1× PBS, 0.05% Tween-20), incubated with His-COP1 for 45 min at room temperature with shaking (1100 rpm), and blocked with selection buffer (1× PBS, 0.05% Tween-20, 0.1 mg/mL BSA, pH 7.5). The immobilised protein was incubated with 25 µL of peptide library for 1 h at room temperature with shaking. Typical final conditions were 5 µg His-COP1, 1.25 µL Ni-NTA magnetic beads, and a 25 µL selection volume. Following incubation, beads were washed 3 × 1 mL with 1× PBS to remove unbound peptides. Bound peptides were eluted by heating the beads in 10 µL 1× PBS at 80 °C for 10 min, and eluates were collected after magnetic separation and stored at −20 °C prior to LC–MS/MS analysis.

5.3 Affinity selection with Libraries 3 and 4

Affinity selections were performed using His-COP1 (10 µg per condition) immobilised on HisPur™ Ni-NTA magnetic beads (5 µL, 12.5 mg/mL). Six conditions were evaluated in triplicate, comprising no-protein controls (conditions 1–3) and His-COP1-containing samples (conditions 4–6). Peptide libraries 3 and 4 (262,144 members) were incubated with the beads at a total concentration of 31.46 mM, corresponding to ~120 nM per individual library member (~3 pmol/member in 25 µL), in selection buffer (1× PBS, 0.05% Tween-20, 0.1 mg/mL BSA, pH 7.5). Following protein immobilisation (45 min, room temperature, 1250 rpm) and preparation of beads for selection (incubation with 100ul selection buffer, 5min, room temperature), beads were resuspended in 25ul of Library (3 or 4) prepared in selection

buffer at the specified concentrations. Samples were incubated for 1 h at room temperature with shaking at 1250 rpm. Typical final conditions were 10 µg His-COP1, 5 µL Ni-NTA magnetic beads, and 25 µL selection volume. Beads were washed 3 × 1 mL with 1× PBS, and bound peptides were eluted by heating in 10 µL 1× PBS at 80 °C for 10 min. Eluates were collected following magnetic separation and stored at −20 °C prior to LC–MS/MS analysis.

6. Identification of Library Hits and analysis

6.1 LC-MS/MS analysis of Library 1-4

Samples were injected onto a 0.3 x 10 mm trap column packed with 3 µm 300A C4 media (Dr Maisch, Ammerbuch-Entringen, Germany) and desalted for 2 minutes at 20 µl/min, before being separated with the following gradient at 6 µl/min using a Micro M5 UPLC system (AB SCIEX, Framingham, MA, USA): 0 min 15% B; 5.9 min 97% B; 7.9 min 97% B; 8 min 15% B; 10 min 15% B, where A was 0.1% formic acid in water and B was 0.1% formic acid in MeCN. The LC effluent was directed into a ZenoTOF 7600 Quadrupole-Time-of-Flight mass spectrometer (AB SCIEX) for Information Dependent Analysis (Data Dependent Analysis, DDA), comprising a TOF-MS scan from 350-1500 m/z for 100 ms, followed by MS/MS scans (m/z 100-1500) on the 10 most abundant multiply-charged features for 40 ms each, giving a total cycle time of 0.57 seconds. The mass spectrometer and UPLC system were under the control of the Sciex OS software package (AB SCIEX).

6.2 Precision-recall analysis

De novo candidate sequences were analysed in line with the method reported by Vinogradov.[2] First, working with a list of all peptide candidates, sequences not matching library design rules (sequence length, C-terminal Lysine, correct monomers in every position) were eliminated. Unique sequences were determined by removing redundant oxidised methionine containing sequences, and those with lower ALC scores. The proportion of correct sequences from the library (recall) was plotted against the proportion of correct sequences in the total ID list (precision) at different ALC scores (0, 30, 50, 70, 80,90, 95).

6.3 Data Processing and Analysis of Peptide Libraries with FragPipe

Raw LC–MS/MS data were processed using FragPipe (Nesvilab, version 22.0) employing the label-free quantification with match-between-runs (LFQ-MBR) workflow. Raw .wiff files were converted to .mzML format using MSConvert (ProteoWizard version 3.0) with peak picking enabled.
Custom FASTA databases were generated from combinatorial peptide libraries using PeSA (version 2.2). Decoy sequences were generated by sequence reversal and incorporated into the databases using the prefix “rev_”. For Libraries 3 and 4 contaminant proteins including BSA and COP1 were included in the FASTA files.

Database searching was performed using MSFragger with no enzyme specificity, precursor mass tolerance of ±20 ppm, fragment mass tolerance of 0.02 Da, peptide lengths of 7–9 amino acids, and a peptide mass range of 500–5000 Da and split database enabled (set to

“1”) for Libraries 2–4. For libraries 2 and 3, methionine oxidation (+15.99 Da) was included as a variable modification, while C-terminal amidation (−0.984 Da) was specified as a fixed modification. For Library 4, non-canonical amino acids were represented by substituted canonical residues with defined mass offsets:

**Non-canonical amino acid**	**Replaced with amino acid**	**Mass Difference**
Fmoc-D-Ala-OH	A	0
Fmoc-1-amino-1-cyclopropane carboxylic acid	U	-67.9165
Fmoc-L-Propargyglycine	C	-7.972
Fmoc-D-Dab(Boc)-OH	T	-0.984
Fmoc-L-β-t-butylalanine	N	13.0568
Fmoc-D-Cit-OH	R	0.984
Fmoc-D-3-(2-Naphthyl)-alanine	W	11.0047
Fmoc-Lys(Z)-OH	O	24.984

Peptide and protein identifications were filtered to a 1% false discovery rate (FDR). Results were further validated using Percolator with a probability threshold of 0.5. Quantification was performed using IonQuant with default LFQ and match-between-runs settings enabled.

6.4 Post-sequencing analysis

Following peptide sequencing, the list of all identified peptides was exported as .csv files and further analysed and visualised using Perseus (version 2.0.10.0). Peptides with a false-discovery rate above 1% were excluded. The list of peptides was refined by summing MS1 intensity (area under peak curve) values across replicates from the target experimental conditions (COP1), and subtracting from this the summed intensity values present in the replicates of the no-protein control. Peptides with summed intensity values of 0 or less were removed. Quantitative data were visualised with rank-ordered waterfall plots using Graph Pad Prism (version 10) to identify top hits.

6.5 Hit-Triage analysis

Affinity selection experiments were performed in duplicate, and sequences identified in both replicates, or those containing VP motifs were considered for further hit resynthesis and validation.

7. Hit Validation

7.1 Surface Plasmon Resonance (SPR) assay of selected Hit peptides

SPR experiments were performed on a Biacore T200 using a Series S Sensor Chip NTA (SCBS NiHC1000M). The experiments were conducted in HBSTE buffer (10 mM HEPES pH 7.4, 150 mM NaCl, 0.05% (v/v) Tween-20, and 50 µM EDTA). All experiments were

conducted at 10 °C. The sensor surface was conditioned with 20 mM EDTA (25 µL/min) and charged with $NiCl_2$ (15 µL/min) prior to capture of His-COP1 (0.2 µM) on flow cell 2 (FC2) at 5 µL/min to approximately 1000 Response Units (RU). Flow cell 1 (FC1) served as the reference surface.

Peptide hits from Libraries 1–4 were analysed by multi-cycle kinetics. Peptides were injected as an 11-point concentration series (0–100 µM) at 30 µL/min, with defined association and dissociation phases for each injection. Positive control peptide TRIB1 (**1**) was included in every run for day to day comparisons.

Following each analyte injection, the sensor chip surface was regenerated, recharged with Ni^{2+}, and fresh His-COP1 was recaptured before the next cycle.

Sensorgrams were reference-subtracted (FC1) and baseline-corrected using Biacore T200 Evaluation Software. Processed data were exported in .txt format and analysed in GraphPad Prism (version 10).

Sensorgrams were double blank-subtracted (reference flow cell and vehicle only analyte) using Biacore T200 Evaluation Software. Concentration response data were exported in .txt format and analysed in GraphPad Prism (version 10). Response (RU) was normalised to the theoretical R_{max} which was calculated using the following equation:

$$R_{max} = \frac{MW_{analyte}}{MW_{ligand}} \times R_{ligand} \times Valency$$

Where the analyte was the peptide binder, the ligand COP1 and the valency assumed to be 1. Equilibrium binding constants (K_D) were calculated by fitting a 4-parameter concentration response curve with the upper limit constrained to 100 (representing the response at theoretical R_{max} = 100%.

7.2 Time-resolved Fluorescence resonance energy transfer (TR-FRET) assay of selected Hit peptides

Time-Resolved Fluorescence Resonance Energy Transfer (TR-FRET) assays were performed in white 384-well plates (HSP3805, Bio-Rad) in a total volume of 20 µL per well. Data were acquired using a VANTAstar plate reader to detect FITC (520 nm) and terbium (620 nm) emission signals following excitation at 426 nm (dichroic filter). The detection settings included an integration delay of 60 µs and an integration time of 200 µs. All assays were conducted in Phosphate Buffered Saline (PBS), pH 7.4, 0.5 mM TCEP (Tris(2-carboxyethyl)phosphine hydrochloride), 5% Glycerol, 0.02% Tween-20 assay buffer.

To establish the optimal fitc-TRIB1 peptide concentration for competition assays, 50 nM COP1-WD40 was incubated with 0.2 nM MAb Anti-6HIS-Tb cryptate Gold (61HI2TLF, Revvity). A dilution series of FITC-TRIB1 (349–367) was prepared, ranging from 10 µM down to 313 nM. Resulting raw readings were turned into 520:620 ratio's in excel and were analyzed using GraphPad Prism 10.6.1.

For the competition experiments, 50 nM COP1-WD40 and 350 nM FITC-Trib1 peptide were added to the 384-well plate. Competing peptides were serially diluted 3-fold in binding buffer prepared in a three-fold dilution series ranging from 500 µM down to 8.5 nM, including a buffer-only control. The competing peptides were added to the protein-probe mixture, mixed, and centrifuged at 3000 rcf for 5 minutes. The plate was then incubated for 30 minutes at room temperature in the dark. Finally, MAb Anti-6HIS-Tb was diluted and added to each well. After a second centrifugation step, TR-FRET measurements were recorded at 0, 60, 120, and 180 minutes.

Fifty percent inhibition values (IC_{50} values) were calculated by fitting a 4-parameter concentration response curve with the upper limit constrained to 100 (representing the TR-FRET signal with protein only) and the lower limit constrained to 0 (representing the signal with no protein to mimic full inhibition).

References

(1) Kaiser, E.; Colescott, R. L.; Bossinger, C. D.; Cook, P. I. Color Test for Detection of Free Terminal Amino Groups in the Solid-Phase Synthesis of Peptides. *Anal. Biochem.* **1970**, *34* (2), 595–598.

(2) Vinogradov, A. A.; Gates, Z. P.; Zhang, C.; Quartararo, A. J.; Halloran, K. H.; Pentelute, B. L. Library Design-Facilitated High-Throughput Sequencing of Synthetic Peptide Libraries. *ACS Comb. Sci.* **2017**, *19* (11), 694–701.
